# SkitoSnack 2.0 - A Bloodmeal Alternative for *Anopheles* and *Aedes* Mosquitoes

**DOI:** 10.1371/journal.pntd.0014188

**Published:** 2026-04-17

**Authors:** Anjali Karki, Hailey A. Luker, Naga Narendra Reddy Potlapalli, F. Omar Holguin, Meenakshi Berwal, Patricia V. Pietrantonio, Immo A. Hansen

**Affiliations:** 1 Department of Biology, New Mexico State University, Las Cruces, New Mexico, United States of America; 2 Department of Plant and Environmental Sciences, New Mexico State University, Las Cruces, New Mexico, United States of America; 3 Department of Entomology, Texas A&M University, College Station, Texas, United States of America; 4 Texas A&M AgriLife Research and Extension Center, Lubbock, Texas, United States of America; University of Florida, UNITED STATES OF AMERICA

## Abstract

SkitoSnack, an artificial blood-meal alternative, was developed in 2018 for rearing *Aedes aegypti* mosquitoes in laboratory culture. This artificial blood-meal diet has a long shelf life and can effectively support the long-term rearing of *Ae. aegypti*. However, *Anopheles* mosquitoes often do not engorge on it. Therefore, in this study we optimized the SkitoSnack recipe for rearing *Anopheles stephensi* mosquitoes. We added, removed or changed individual components from the original recipe and measured engorgement rates, egg numbers, and hatching rates. We identified a new recipe that can effectively support the continuous rearing of *An. stephensi.* We then tested this new diet with *Ae. aegypti* and found engorgement rates, egg numbers, and hatching rates were not statistically different from those of blood-fed females. Using a modified FlyPAD feeding system, we showed that *Ae. aegypti* ingested significantly larger meal volumes of the new diet compared to bovine blood, showing a strong preference for it. Our findings support that our new diet is an effective blood-meal alternative for the rearing of both *Anopheles* and *Aedes* mosquitoes. We named the new recipe SkitoSnack 2.0.

## Author summary

Mosquitoes are the deadliest animal in the world to humans. Counterintuitively, to lessen the harm of mosquitoes, scientists need to grow large numbers of mosquitoes in laboratories for research and for certain population control strategies. Laboratory rearing of mosquitoes requires a continuous supply of fresh animal blood. Currently, fresh blood is supplied either by using live animals or through artificial feeding systems. Animal blood can be expensive and difficult to handle. To address this problem, a blood-meal alternative called SkitoSnack was developed for the yellow fever mosquito, *Aedes aegypti*. Here, we present an improved recipe, SkitoSnack 2.0, that is suitable not only for *Ae. aegypti* but also for *Anopheles stephensi*, a malaria vector. To test this new recipe, we measured feeding rates, egg numbers, and larval hatch rates of *An. stephensi* grown on this diet for five generations. We then compared the metabolites in eggs from mosquitoes fed on either blood or SkitoSnack 2.0 and found that they were nearly identical. We then tested SkitoSnack 2.0 on *Ae. aegypti* and determined that this diet is suitable and that it was even preferred over blood. Our study shows that SkitoSnack 2.0 is a suitable blood-meal alternative for rearing both *An. stephensi* and *Ae. aegypti* mosquitoes.

## Introduction

*Anopheles* mosquitoes are the primary vector of malaria, the deadliest vector-borne disease affecting millions of people annually. The World Malaria Report by the World Health Organization (WHO) in 2023 reported the significant burden posed by malaria worldwide, with 263 million malaria cases and 597,000 deaths [1]. At present, insecticides are the most widely used tactic for controlling vector populations in malaria-endemic regions [2,3]. The excessive use of insecticides results in the development of insecticide-resistant mosquito populations. Therefore, there is a need for the development of novel vector control strategies, like the sterile insect technique, incompatible insect technique [4], gene drive technology [5], or attractive toxic sugar baits [6]. All of these innovative vector control techniques rely on the continuous supply of laboratory-reared mosquitoes for testing, standardization, and release [7]. Blood meal substitutes for rearing hematophagous *Aedes, Anopheles*, and *Culex* species have been developed and tested since the 1950s (Table 1) [8]. However, limited success has been reported on blood meal substitutes that can support multiple species.

**Table 1:** Various bloodmeal substitute diets for mosquito rearing.

Work cited	Name of substitute	Mosquito species	Components of Diet
**[9]**	Simple Alternate meal (AM)	*Ae. aegypti* (Orlando strain)	Spray-dried porcine blood (SDPB), whey protein powder, and 10X Phosphate buffered saline (PBS)
**[10]**	Protein Rich Sugar Solutions (PRSS)	*Anopheles darlingi* (Root)	Bovine serum albumin (BSA) and sucrose solution with or without supplemented salts (NaCl & NaHCO3); BSA (200 OR 400 mg/ml
**[11]**	BLOODless diet	*Anopheles coluzzii* (former *An. gambiae* M form)	(r-liquid diet) enriched artificial meals containing BSA, ATP, cholesterol, amino acids, peptides, and salts
**[3,12]**	SkitoSnack	*Ae. aegypti* (L.) and *An. stephensi*	Bovine serum albumin (BSA), hemoglobin, chicken yolk, glucose, ATP & bicarbonate salts mixtures
**[13]**	Plasma ATP diet	*Ae. aegypti* and *An. gambiae*	Adenosine triphosphate (ATP) supplemented blood plasma diet
**[14]**	Substitute Bloodmeal (SBM)	*Ae. aegypti* (Red Eye strain)	Albumin, hemoglobin, γ-globulin, Tyrod’s supplemented with Cholesterol, Asolectin/ Phosphatidylcholine, and LDL (low density lipoprotein)
**[15]**	A simple protein formulation	*Aedes albopictus* (Skuse)	Bovine serum albumin (BSA) dissolved in Phosphate buffered saline (PBS) solution (NaCl, Na2HPO4, KH2HPO4, and KCl) with ATP as a phagostimulant
**[16]**	Artificial Blood Formulation	*Culex quinquefasciatus*	Ovalbumin, soya infant formula, globulins, and adenosine triphosphate (ATP), salts
**[17]**	Optimization of Kogan (1990)	*Ae. aegypti, Anopheles arabiensis,* and *An. stephensi*	Optimization of Kogan (1990); bovine albumin, hemoglobin, and globulin in a Ringer-based solution, plus ATP as a phagostimulant
**[18]**	A formulated protein diet	*Ae. aegypti* (L.)	Simple mixture of proteins (gamma (γ) globulins, albumin, hemoglobin) with salt and adenosine triphosphate (ATP)
**[19]**	N/A	*Ae. aegypti*	Human serum albumin, human gamma-globulin, hemoglobin, egg albumin, pepsin, lipase, trypsin, proteose-peptone, and the enzymatic hydrolysates of lactalbumin, casein, soybean meal, and yeast
**[20]**	N/A	*Ae. aegypti* and *Anopheles quadrimaculatus*	Skimmed milk and honey

SkitoSnack was developed by Gonzales *et al.* in 2018 as an artificial blood meal alternative for rearing *Ae. aegypti* mosquitoes. It is composed of bovine serum albumin (BSA) as the source of essential amino acids, adenosine triphosphate (ATP) as a phagostimulant, bovine hemoglobin as an iron source, carbohydrates as a source of energy, chicken yolk as a source of cholesterol, and bicarbonate salt mixtures to mimic the pH in animal blood. The effectiveness of SkitoSnack was evaluated based on the following standards: (1) the meal must result in fully engorged females, (2) the meal must support mosquito vitellogenesis, (3) the meal must support large batches of viable eggs, and (4) the meal must generate healthy progeny. With *Ae. aegypti*, SkitoSnack produced satisfactory results in all four categories. In a follow-up study, Kandel and colleagues, in 2020, tested SkitoSnack on multiple strains of *Aedes* mosquitoes over multiple generations and showed that the progeny from SkitoSnack-raised females have similar reproductive performance and life history traits as bovine blood-fed counterparts. However, preliminary testing in our laboratory found that *Anopheles gambiae* mosquitoes did not feed on this SkitoSnack diet. These females probed at the feeder but did not engorge any of the meal.

Therefore, in this study, we optimized the SkitoSnack recipe for rearing *Anopheles* mosquitoes. Optimization was accomplished by changing single components or concentrations of particular components of the original recipe, followed by feeding assays. In the new SkitoSnack 2.0 recipe, hemoglobin was replaced by iron (II) fumarate. We demonstrated that the new recipe can support both *Anopheles* and *Aedes* mosquitoes.

## Materials and methods

### Mosquito strains and rearing

We used two species of mosquitoes for our study. *An. stephensi* STE2 eggs were provided by Dr. Jiannong Xu’s laboratory at New Mexico State University. *Ae. aegypti* eggs of the Liverpool strain were provided by the NIH/NIAID Filariasis Research Reagent Resource Center for distribution through BEI Resources, NIAID, NIH: *Ae. aegypti*, Strain Black Eye Liverpool, Eggs, NR-48921. Mosquito colonies were reared according to previously published protocols [21–23] with few modifications. Approximately 500 eggs were hatched in pans (32 x 23 x 5 cm) filled with two liters of deionized water, and 300 first-instar larvae were separated into different pans filled with approximately three liters of deionized water to avoid larval overcrowding. *An. stephensi* larvae were raised on a diet of powdered fish food (TetraMine Tropical fish Food, VA) and brewer’s yeast (MP Biomedicals, ThermoFisher Scientific, USA) in a 2:1 ratio until pupation. *Ae.* aegypti larvae were fed *ad libitum* on Special Kitty cat food (Walmart stores, Bentonville, AR, USA) until pupation. Pupae were collected in a container filled with deionized water and placed into BugDorm cages (27.5 x 29.5 x 29.5 cm, BugDorm Company, Taichung, Taiwan). *An. stephensi* adult mosquitoes were fed *ad libitum* on a 10% sugar solution. *Ae. aegypti* adults were fed *ad libitum* on a 20% sugar solution [12,24,25]. Mosquitoes were stored at 27 ± 2°C with 80% relative humidity under a 12 h light-dark cycle. All female mosquitoes used in this study were one week old and mated.

### Optimization of the original SkitoSnack recipe for *Anopheles* mosquitoes

We modified the artificial diet, SkitoSnack [12] by changing single components and/or concentrations from the original recipe (**see** Table 2). The ingredients for each modified artificial diet were combined using a mortar and pestle. Diets were stored in powder form at -20°C until use. Diets were hydrated using deionized water, vortexed, incubated at 42°C for 5 minutes, vortexed again until the diet was completely dissolved, and then used promptly in the feeding assay.

**Table 2:** Composition of the original SkitoSnack and all the modified SkitoSnack.

Chemical Components	Original SkitoSnack	Modified SkitoSnack							
		SkitoSnack (-hemo)	Different ATP concentrations			Iron substitutions			
			1X ATP	2X ATP	3X ATP	Ferric nitrate nonahydrate	Ferric chloride	Ferrous fumarate	Ferrous gluconate
Bovine serum albumin	200 mg/ml	200 mg/ml	200 mg/ml	200 mg/ml	200 mg/ml	200 mg/ml	200 mg/ml	200 mg/ml	200 mg/ml
Chicken yolk	5 mg/ml	5 mg/ml	5 mg/ml	5 mg/ml	5 mg/ml	5 mg/ml	5 mg/ml	5 mg/ml	5 mg/ml
Adenosine triphosphate	1.65 mg/ml	1.65 mg/ml	1.65 mg/ml	3.3 mg/ml	4.95 mg/ml	1.65 mg/ml	1.65 mg/ml	1.65 mg/ml	1.65 mg/ml
Glucose	50 mM	50 mM	50 mM	50 mM	50 mM	50 mM	50 mM	50 mM	50 mM
Sodium chloride	150 mM	150 mM	150 mM	150 mM	150 mM	150 mM	150 mM	150 mM	150 mM
Sodium bicarbonate	23 mM	23 mM	23 mM	23 mM	23 mM	23 mM	23 mM	23 mM	23 mM
Potassium chloride	4 mM	4 mM	4 mM	4 mM	4 mM	4 mM	4 mM	4 mM	4 mM
Calcium chloride	2.5 mM	2.5 mM	2.5 mM	2.5 mM	2.5 mM	2.5 mM	2.5 mM	2.5 mM	2.5 mM
Magnesium chloride	0.8 mM	0.8 mM	0.8 mM	0.8 mM	0.8 mM	0.8 mM	0.8 mM	0.8 mM	0.8 mM
Bovine hemoglobin	5 mg/ml	–	–	–	–	–	–	–	–
Ferric nitrate nonahydrate	–	–	–	–	–	0.0005 mg/ml	–	–	–
Ferric chloride	–	–	–	–	–	–	0.2 mg/ml	–	–
Ferrous fumarate	–	–	–	–	–	–	–	0.2 mg/ml	–
Ferrous gluconate	–	–	–	–	–	–	–	–	0.2 mg/ml

ATP (Adenosine triphosphate); SkitoSnack without hemoglobin (-hemo); mM (millimolar); mg/ml (milligram per milliliter)

### Membrane feeding assay

Feeding assays were performed according to previously published protocols by Gonzales *et al.*[23] with a few modifications. Females (n = 25–30) of *An. stephensi* mosquitoes were transferred into smaller cages (15 x 15 x 15 cm, BugDorm Company, Taichung, Taiwan) for feeding. Mosquitoes were sugar-starved for 16 – 18 hours before experiments. For meal assessments, mosquitoes were offered one diet, either defibrinated bovine blood (HemoStat Laboratories, Dixon, CA, USA) as a control or a modified SkitoSnack, formulation in a membrane feeder (Chemglass Life Science, CG-1835, Vineland, NJ, USA) heated to 37°C. All feeding experiments were performed in the mornings for 60 min. Un-engorged females were subsequently removed from the cage with an insect aspirator. A total of four biological replicates were performed for each formulation. After each feeding trial, three main biological parameters were evaluated to select the suitable modified SkitoSnack recipe: 1) engorgement rate, 2) number of eggs produced (fecundity), and 3) number of larvae emerged from eggs (fertility). The same procedures were performed for *Ae. aegypti*.

### Engorgement rates

The number of engorged female mosquitoes after feeding on each diet was counted using a handheld counter. Engorged females were identified based on engorgement status scores described by Pilitt and Jones [26]. Engorgement status was determined visually. We assigned status scores ranging from 0 to 5. The scores are based on the degree of distension of the abdomen. Stage 0 indicates an unfed mosquito with a shrunken abdomen. Stage 1 females showed the ventral plates form a flattened surface under the curved top of the abdomen. Stage 2 is identified by a completely cylindrical abdomen when viewed from any angle. Stage 3 is assigned when the side view of the abdomen is clearly divided into dorsal and ventral sections by the pleural membrane. Stage 4 is characterized by the moderate dorsolateral expansion of the abdomen with a visible pleural membrane. Stage 5 is characterized by the pronounced dorsolateral expansion of the abdomen with a large amount of pleural membrane visible. We counted any mosquito with the qualitative engorgement status of “Stage 4” and “Stage 5” as engorged. The engorgement rate was calculated by dividing the number of engorged females by the total number of female mosquitoes in each cage.

To assess the effect of different diets on female mosquitoes’ feeding rate, we used an unpaired t-test while comparing two diet groups and a one-way ANOVA test followed by Tukey’s post hoc test for multiple comparisons while comparing three or more diet groups. The dependent variable was the engorgement rate, and the independent variable was the type of diet, which included defibrinated bovine blood and all the modified SkitoSnack formulations, as shown in Table 2. During analysis, Tukey’s post hoc test was used for multiple comparisons between diet groups.

### Egg deposition and hatching rates

For *An. stephensi*, a moist filter paper (Whatman qualitative filter paper, Grade 5, 1005–150, Sigma-Aldrich) was added 48 h and 72 h post-meal for *en masse* egg deposition for 24 h. The filter papers were respectively removed, 72 h and 96 h later, and eggs were counted manually using a stereo zoom microscope (Leica S6D, North Central Instruments). Immediately after counting, the eggs were transferred to a pan for hatching. The number of first- and second-instar larvae in the pans was counted 4–5 days after hatching. The hatch rate was calculated by dividing the number of larvae by the total number of eggs hatched. The same procedure was performed for *Ae. aegypti*, except a damp seed-germination paper (Manufacturer: Ahlstrom-Munksjo, Product Number: SB39211) was used for egg deposition, and eggs were allowed to desiccate for one week after counting.

To determine the effects of different diets on egg production and egg viability, we performed an unpaired t-test while comparing two diet groups and a one-way ANOVA test with Tukey’s post hoc test for multiple comparisons while comparing three or more diet groups. The dependent variables were the number of eggs laid and the hatch rate. The independent variable was the type of diet, which consisted of defibrinated bovine blood and all the modified SkitoSnack formulations shown in Table 2. Tukey’s post hoc test was used for multiple comparisons between different diet groups.

### Adult mosquito body size measurement

Wing length and body weight were measured following the published protocols by Gonzales *et al.* [12] and Zuharah *et al.* [27]. A random sample of 31 male and 31 female mosquitoes was used for wing measurement from both bovine blood and SkitoSnack 2.0-raised mosquitos. Mosquitoes were starved for 16–18 hours.

Then, all the mosquitoes were ice anesthetized for 60 s, and the right wing of each mosquito was detached for the measurement. The wing lengths of each mosquito were determined using a stereo microscope and a micro scale (S1 stage micrometer, PYSER-SGI, United Kingdom). To obtain the average mosquito weight, the weight of a different set of 33 male and 33 female mosquitoes was measured to 0.1 mg using an XPR micro balance (Mettler Toledo, Columbus, OH). A total of three biological replicates were performed for both the wing measurements and weights.

Wing lengths and body weights were compared among the male and female mosquitoes reared on the different diets using a one-way ANOVA with Tukey’s post hoc test. The dependent variables were wing length or body weight, whereas the independent variable was the type of diet, which included defibrinated bovine blood and SkitoSnack 2.0. Tukey’s post hoc test was used for multiple comparisons between the diet groups.

### Mosquito egg metabolomics

Mosquito eggs’ total metabolites were extracted and analyzed as described by Gonzales et al [12] with a few modifications. Eggs were collected from two cohorts of *An. stephensi* mosquitoes (1) *An. stephensi* fed on defibrinated bovine blood, and (2) *An. stephensi* fed on SkitoSnack 2.0. Approximately 8–10 mg of eggs were weighed and transferred into 1.7 mL microcentrifuge tubes. Sample preparation for gas chromatography/ mass spectrometry (GC/MS) was performed using Varian Saturn 2000 (Varian Inc., Walnut Creek, CA, USA). The details and specifications for sample preparation are provided in **S2 File**. Data was uploaded to MS-DIAL [28] software for automated peak deconvolution, alignment, and metabolite annotation based on fragmentation pattern and retention indices. Peak area values were normalized to the sample weight and internal standard (Ribitol) peak area. Data were log_2_-transformed for statistical analysis.

### FlyPAD feeding bioassays

The automated flyPAD feeding system was used as described in a previously published protocol by Henriques-Santos et al. [29]*,* with a few modifications. The flyPAD system consists of twelve chambers, each with four arenas, and each arena with two wells. The chambers were placed on two slide warmers (Barnstead/Lab-Line, USA, and Premiere, XH-2001, C&A Scientific Co., Inc.), maintained at 39°C. In choice feeding assays, three microliters of bovine blood and SkitoSnack 2.0 were pipetted into each well of the arena. An individual female was then placed in the arena to record their feeding behavior for 30 min.

The flyPAD system recorded nine feeding behavioral variables, including number of sips (contact of proboscis with food), sip duration (period of contact of proboscis with meal) and their intervals; number, duration, and intervals of feeding bursts (three or four consecutive sips), feeding bouts (three or four consecutive bursts) [30] using the Bonsai data stream processing package [31]. A Blackfly camera (FLIR Integrated Imaging Solutions, Inc., BFS-U3-16S2C-CS) was used to capture video recordings of a sample arena, and MATLAB (MathWorks Inc., Portola Valley, CA, USA) was used for all signal processing and data analysis. Besides the nine feeding variables, the software also provided cumulative feeding data, which included the average number of sips taken by all females, recorded every 10 seconds throughout the 30-minute recording period. The cumulative preference index (PI) was calculated using the formula: PI = (n_BB_ - n_SS_)/ (n_BB_ + n_SS_), where n_BB_ and n_SS_ represent the cumulative number of sips measured for bovine blood and SkitoSnack 2.0 meal, respectively, at consecutive 10 seconds intervals, for the 30 minutes of recordings [30]. The details and specifications regarding meal preparation and meal volume quantification for choice and non-choice feeding assays are provided in S3 File.

All variables measured by the automated FlyPAD feeding system were analysed using the Mann-Whitney U test, whereas the cumulative feeding behavioral data were analysed using a two-way ANOVA with repeated measures.

The dependent variables were the nine feeding behavioral parameters, such as number of sips, sip duration, intersip intervals, number of feeding bursts, feeding burst duration, interburst intervals, number of activity bouts, activity bout duration, and interbout intervals. The independent variable was the type of diet, which included defibrinated bovine blood and SkitoSnack 2.0. A two-way ANOVA followed by multiple comparisons of the means was used to identify significant differences in feeding behavior across multiple time points. The Mann-Whitney U test was used to analyze individual feeding parameters.

### Statistical analysis

All statistical analyses were performed using GraphPad Prism 9 (GraphPad Software, San Diego, CA) with a significance level of α ≤ 0.05. All the data from membrane feeding assays were tested for normal distribution using the Shapiro-Wilk normality test. Normality of the residuals was tested using Q-Q plots. *An. stephensi* engorgement, egg number, and egg hatching data followed a normal distribution, so an unpaired t-test was used to compare two diet groups, and a one-way ANOVA test followed by Tukey’s post hoc test was used to compare three or more diet groups for multiple comparisons. Likewise, *Ae. aegypti* engorgement, egg number, and hatching data also followed a normal distribution, so an unpaired t-test was used. All the variables measured by the FlyPAD feeding system were analyzed by the Mann-Whitney U test. The p-values obtained from Mann-Whitney U tests for FlyPAD feeding variables were adjusted for multiple comparisons using the Holm-Sidak method. Adjusted p-values were compared against α = 0.05 to determine significance. The cumulative feeding behavioral data were analyzed using a two-way ANOVA with repeated measures and multiple comparisons of means between groups at each time point. All the raw data for this study are available in the S1 File.

## Results

### Female reproductive performance after feeding on different meals

**Fig 1:**
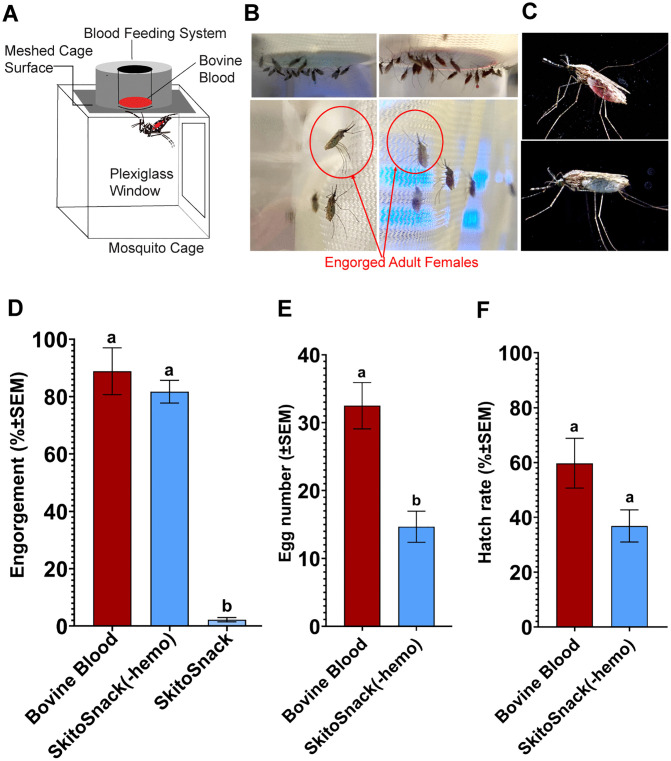
Improved reproductive success after hemoglobin removal from SkitoSnack. Female reproductive performance after feeding on bovine blood, original SkitoSnack or modified SkitoSnack diets. **(A)** Membrane feeding assay set-up. **(B)** Photo of female *Anopheles stephensi* fed on SkitoSnack without hemoglobin(-hemo) (**left**) and bovine blood (**right**) using a water-jacketed glass membrane feeder. **(C)** Photo of adult females fully engorged on bovine blood (**top**) and SkitoSnack(-hemo) (**bottom**). **(D)** Mean percent engorgement on different diets. **(E)** Mean number of eggs laid per engorged female. **(F)** Mean percent egg hatch rates. A one-way ANOVA followed by Tukey’s multiple comparison test was used to analyze the statistical significance among different diet groups in subfig **D** (P < 0.0001). An Unpaired t-test was used to determine the significant differences between diet groups in subfigs **E** & **F** (P < 0.05). Columns with different letters indicate a significant difference. Photographs in (**B**) and (**C**) were taken by the author (**AK**).

The reproductive performance of *An. stephensi* on bovine blood, original SkitoSnack and modified SkitoSnack diets were compared (Fig 1). The schematic set-up of the membrane feeding system is shown in Fig 1A, where *An. stephensi* females were fed on different diets. Fig 1B shows the engorged females fed on bovine blood and the SkitoSnack without hemoglobin(-hemo) diet, and detailed pictures of the respective females are in Fig 1C.

The engorgement rate was significantly different among the three diet groups (F _(2,9)_ = 83.67, P < 0.0001). *An. stephensi* females fed on the original SkitoSnack diet had significantly lower engorgement rates (2.25 ± 0.62%) compared to bovine blood (88.87 ± 8.1%) and the SkitoSnack(-hemo) (81.73 ± 3.9%) diets. However, engorgement rate was similar between the bovine blood-fed and the SkitoSnack(-hemo)-fed groups (Fig 1D, P = 0.618). The mean number of eggs laid was significantly different between the females fed on bovine blood and the SkitoSnack(-hemo) (t_6_ = 4.33, P < 0.005). Females fed on bovine blood laid significantly more eggs (33 ± 3.42 eggs) compared to those fed on SkitoSnack(-hemo) (15 ± 2.3 eggs). However, *An. stephensi* females offered the original SkitoSnack diet deposited no eggs (**Fig 1E**). The hatch rate of the eggs laid by the females fed on SkitoSnack(-hemo) and bovine blood diets was not significantly different (t_6_ = 2.12, P = 0.08). The mean hatch rate was 36.85 ± 5.8% in the SkitoSnack(-hemo) group and 59.73 ± 9.0% in the bovine blood fed group (**Fig 1F**).

### The impact of different iron supplements in the modified SkitoSnack diet on egg deposition

**Fig 2:**
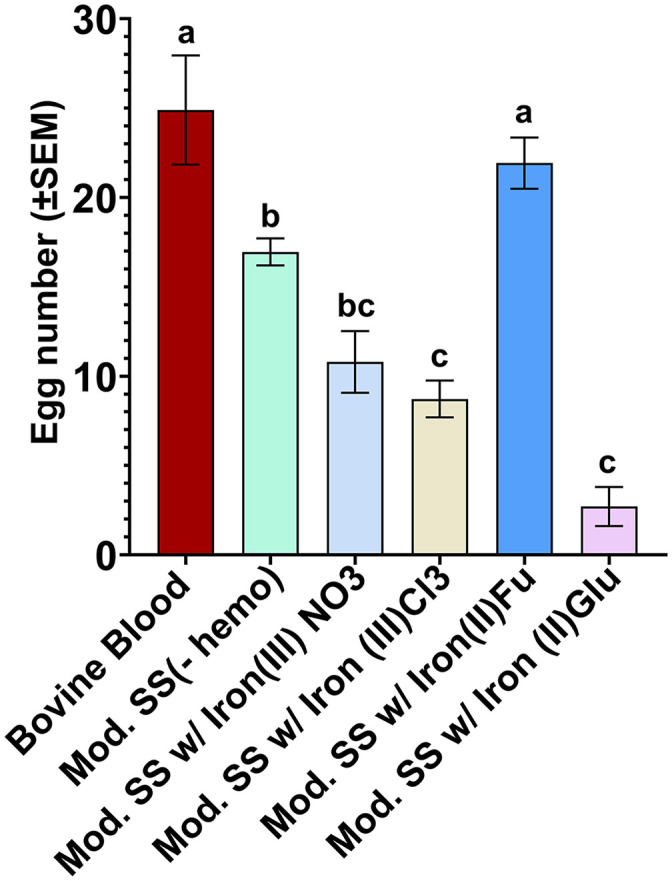
Restored fecundity after addition of iron (II) fumarate to SkitoSnack. Shown are the mean number of eggs laid per female after supplementing modified SkitoSnack with different iron sources. Meals were bovine blood, modified SkitoSnack without Iron, or SkitoSnacks with non-heme iron substitutes for hemoglobin (iron (III) nitrate nonahydrate, iron (III) chloride, iron (II) fumarate, and iron (II) gluconate). “Mod. SS” represents modified SkitoSnack. A one-way ANOVA with Tukey’s multiple comparison test was used to analyse the statistical significance among different diet groups. Columns with different letters indicate a significant difference (P < 0.0001)*.*

To improve the egg numbers laid by females fed on modified SkitoSnack, we tested four different non-heme iron supplements: iron (III) nitrate nonahydrate, iron (III) chloride, iron (II) fumarate, and iron (II) gluconate. The mean number of eggs laid was significantly different among the modified SkitoSnack diet (F _(5,18)_ = 24.85, P < 0.0001). Supplementation of iron (III) chloride and iron (II) gluconate significantly decreased egg numbers with 8.73 ± 1.03 and 2.71 ± 1.1 eggs per female, respectively, compared to modified SkitoSnack without hemoglobin (16.96 ± 0.75). Supplementation with Iron (III) nitrate nonahydrate resulted in similar egg numbers (10.81 ± 1.72) compared to modified SkitoSnack without hemoglobin. Significantly, however, females fed on modified SkitoSnack with Iron (II) fumarate laid a higher egg number (21.93 ± 1.43) that was also similar to that of females fed on bovine blood (24.90 ± 3.04) (Fig 2).

### SkitoSnack 2.0 recipe

To optimize the original SkitoSnack recipe for the rearing of *An. stephensi,* we removed bovine hemoglobin, tested different ATP concentrations, and tried different iron sources. The removal of hemoglobin from the original SkitoSnack recipe improved engorgement rates but resulted in reduced egg numbers. We then tested different ATP concentrations to increase *An. stephensi* engorgement. We tested ATP at concentrations of 3, 6, and 9 mM. However, different ATP concentrations had no significant impact on the number of engorged females (see S2 File, S1 Fig; P = 0.924). The replacement of bovine hemoglobin with iron (II) fumarate significantly improved the number of eggs laid. Fig 2. Based on these findings, the recipe for SkitoSnack 2.0 that was used for all further studies is presented in Table 3.

**Table 3:** Components of original SkitoSnack and SkitoSnack 2.0.

Components	Original SkitoSnack	SkitoSnack 2.0	Company-Product	CAS number
Bovine serum albumin	200 mg/ml	200 mg/ml	Research Products Intl.-A30075	9048–46–8
Bovine hemoglobin	5 mg/ml	–	Sigma Aldrich-H3760	9008-02-0
Ferrous fumarate	–	0.2 mg/ml	Sigma Aldrich-F5381	141-01-5
Chicken yolk	5 mg/ml	5 mg/ml	Sigma Aldrich-E0625	N/A
Adenosine triphosphate	1.65 mg/ml	1.65 mg/ml	Sigma Aldrich-A2383	34369-07-8
Glucose	50 mM	50 mM	Sigma Aldrich-G7021	50-99-7
Sodium chloride	150 mM	150 mM	Sigma Aldrich-S7653	7647-14-5
Sodium bicarbonate	23 mM	23 mM	Sigma Aldrich-S6297	144-55-8
Potassium chloride	4 mM	4 mM	Sigma Aldrich-P9333	7447-40-7
Calcium chloride	2.5 mM	2.5 mM	Sigma Aldrich-C1016	10043-52-4
Magnesium chloride	0.8 mM	0.8 mM	Sigma Aldrich-M8266	7786-30-3

### Reproductive performance of *An. stephensi* fed on SkitoSnack 2.0

**Fig 3:**
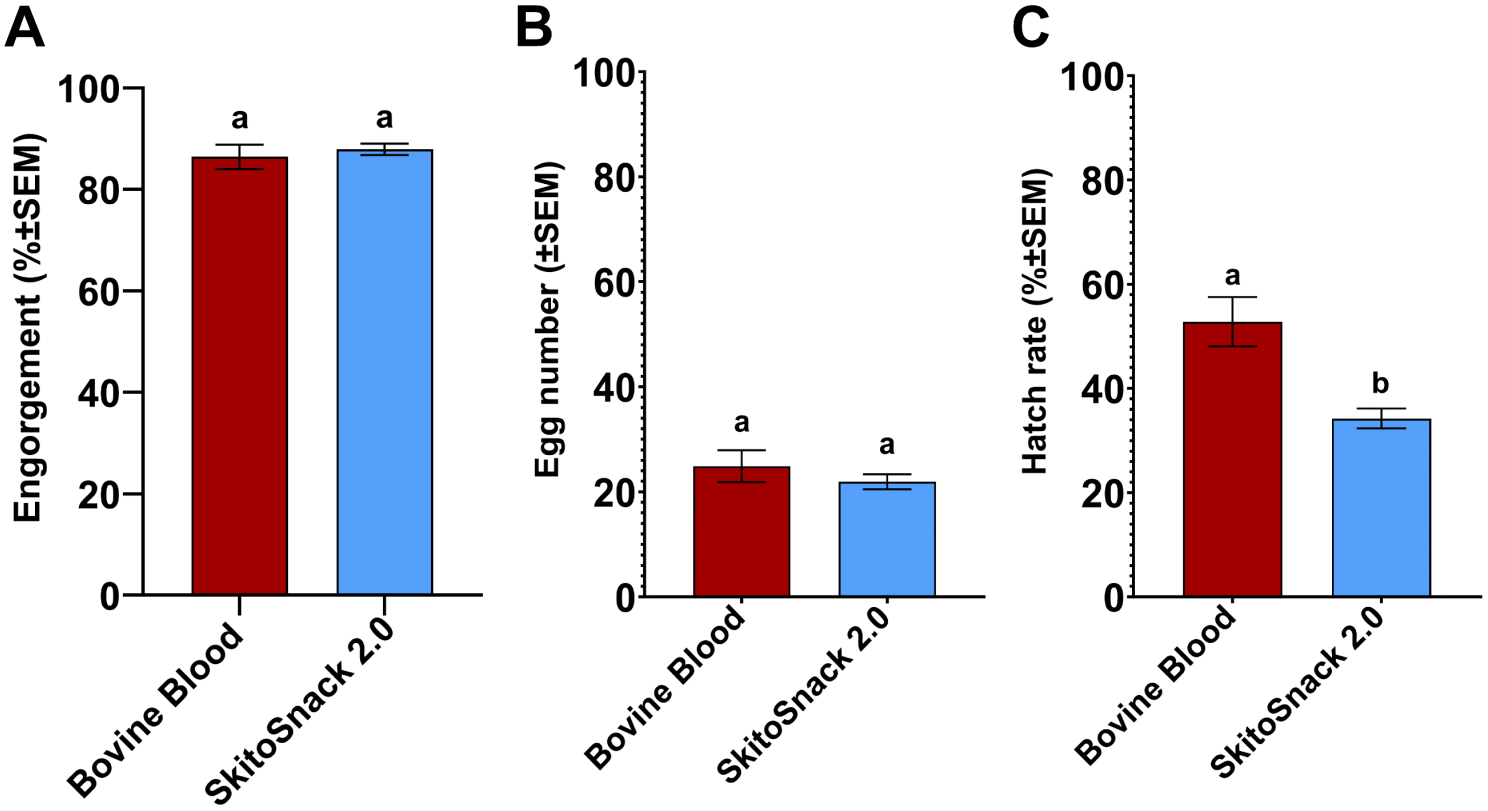
SkitoSnack 2.0 can replace vertebrate blood in *An. stephensi* culture. Reproductive performance of female *An. stephensi* fed on bovine blood or SkitoSnack 2.0. **(A)** The mean percent engorgement rate of females offered bovine blood or SkitoSnack 2.0. **(B)** The mean number of eggs laid per female engorged on bovine blood or SkitoSnack 2.0. **(C)** The mean percent larval hatch rates. An Unpaired t-test was used to determine the significant differences between diet groups. Columns with different letters indicate significant difference (P < 0.05). SEM stands for standard error of the mean.

We tested the reproductive performance of *An. stephensi* fed on the new SkitoSnack recipe, SkitoSnack 2.0. We found similar engorgement rates, 87.93 ± 1.12% and 86.43 ± 2.41%, (**Fig 3A**; t_6_ = 0.563, P = 0.594), and egg numbers, 21.93 ± 1.43 and 24.90 ± 3.04, (**Fig 3B**; t_6_ = 0.883, P = 0.411), between female mosquitoes fed on SkitoSnack 2.0 and bovine blood, respectively. However, larval hatching rates differed significantly, being lower in the SkitoSnack 2.0-fed group compared to the bovine blood-fed group, 34.25 ± 1.9% and 52.81 ± 4.72%, respectively (**Fig 3C**; t_6_ = 3.64, P < 0.011).

### Reproductive performance and size of *An. stephensi* reared on SkitoSnack 2.0 for five generations

We reared *An. stephensi* mosquitoes for five generations exclusively on a SkitoSnack 2.0 diet and measured the engorgement rate, number of eggs laid, and larval hatch rates.

**Fig 4:**
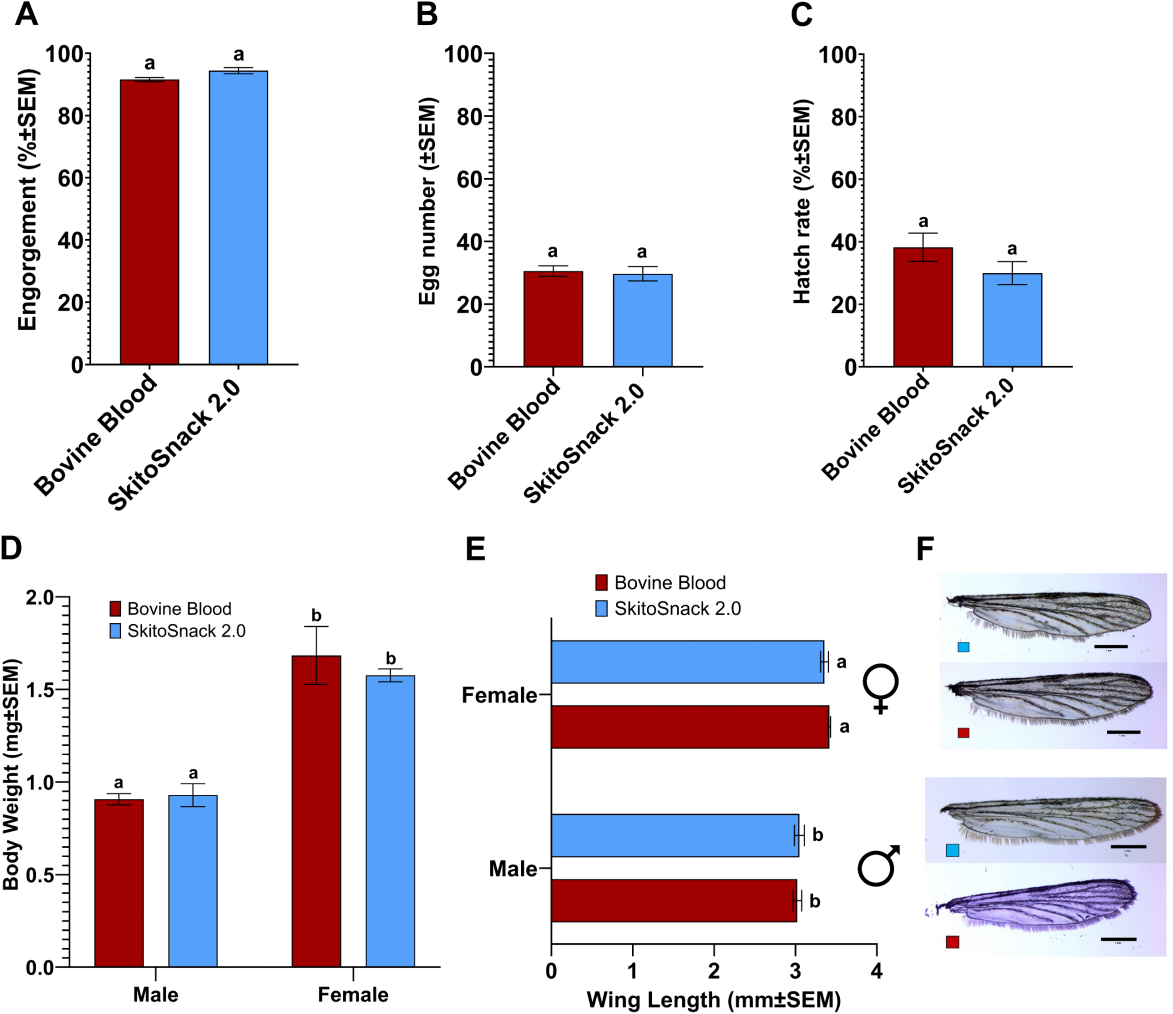
*An. stephensi* mosquitoes raised for five generations on SkitoSnack 2.0 show no phenotypic differences in reproductive performance and size. **(A)** Mean percent engorgement, (**B**) number of eggs laid per engorged female, and (**C**) percent larval hatch rate of fifth generation SkitoSnack 2.0 and bovine blood raised mosquitoes. **(D)** Mean bodyweight and **(E)** Mean wing length of fifth generation SkitoSnack 2.0 and bovine blood raised mosquitoes). An Unpaired t-test was used to determine the significant differences between diet groups in subfigs **A-C** (P < 0.05). A one-way ANOVA followed by Tukey’s multiple comparison test was used to analyze the statistical significance among different diet groups in subfigs **D** & **E** (P < 0.0001). Columns with different letters indicate a significant difference. **(F)** Representative images of the wing of males and females raised on SkitoSnack 2.0 and bovine blood. The scale bar is 1mm. Photographs were taken by the author (**AK**).

Our results show that the engorgement rate was similar between the fifth-generation females raised on two diet groups (t_6_ = 2.37, P = 0.056), with Bovine blood at 91.57 ± 0.67% and SkitoSnack 2.0 at 94.41 ± 0.99% (Fig 4A). Similarly, eggs laid per female (t_6_ = 0.296, P = 0.777) and hatch rates of the eggs (t_6_ = 1.419, P = 0.206) were also similar between the fifth-generation females. The mean eggs laid per female was 29.68 ± 2.28 in the SkitoSnack 2.0 and 30.51 ± 1.68 in the bovine blood-fed group (Fig 4B). The mean hatch rate was 29.98 ± 3.67% in the SkitoSnack 2.0 and 38.25 ± 4.52% in the bovine blood-fed group (Fig 4C).

The mean body weight and wing length of the fifth-generation males and females mosquitoes reared on SkitoSnack 2.0 were not significantly different compared to the bovine blood-reared counterparts (Fig 4D; F _(1,8)_ = 0.2392, P = 0.638; Fig 4E; F _(1,8)_ = 0.137, P = 0.721).

### Mosquito egg metabolomics study

**Fig 5:**
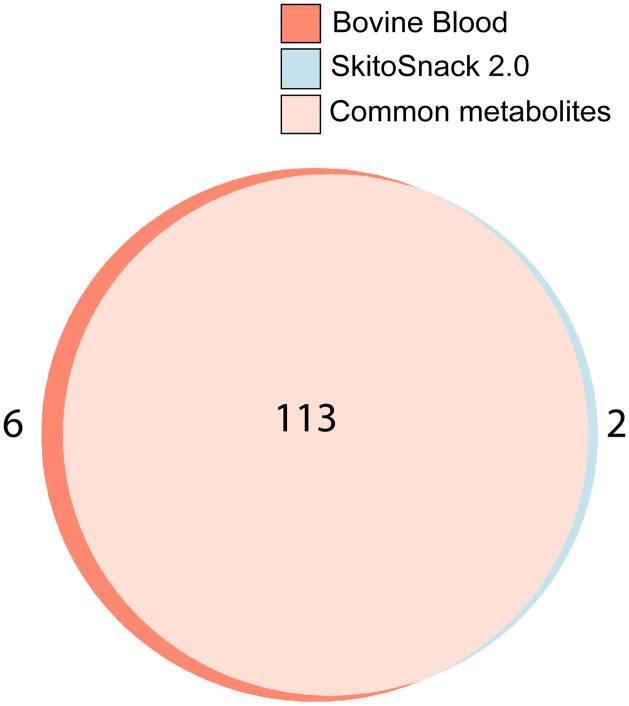
GC/MS metabolite composition profiles of *An. stephensi* eggs.

Untargeted GC-MS metabolomics revealed distinct metabolic profiles between eggs collected from female mosquitoes fed on SkitoSnack 2.0 and those fed on bovine blood (Fig 5). A total of 121 metabolites were detected, with 113 shared across both treatments, while bovine blood and SkitoSnack 2.0 contained six and two unique metabolites, respectively (Fig 5). The six unique metabolites for bovine blood were asparagine, capric acid, diglycerol, dodecanoic acid, Malonic acid, and pentadecanoic acid, whereas two unique metabolites for SkitoSnack 2.0 were lauric acid and isopropanol ester. Principal Component Analysis showed clear clustering and separation between bovine blood and SkitoSnack 2.0 samples, with PC1 explaining 71.5% of the variance and PC2 accounting for 9.1%, indicating significant abundance differences of the metabolites (see S2 File, S2 Fig). Hierarchical clustering of Z-score normalized, log-transformed intensities further confirmed these differences, with distinct metabolite enrichment patterns in each treatment (see S2 File, S3 Fig). Together, these analyses showed that while bovine blood and SkitoSnack 2.0 share a core set of metabolites, they differed in their overall relative abundance profiles.

Proportional Venn diagram illustrating the shared and unique metabolites identified through GC-MS-based untargeted metabolomics between two meal formulations: Bovine blood and SkitoSnack 2.0. The size of each circle is scaled to represent the total number of unique metabolites within that diet group, and the overlapping area shows metabolites shared between groups. The numbers within/ beside the circle represent a specific count of metabolites in each group. A total of 121 unique metabolites were detected, with 113 metabolites shared between both diet groups, six (asparagine, capric acid, diglycerol, dodecanoic acid, malonic acid, and pentadecanoic acid) unique to bovine blood, and two (lauric acid and isopropanol ester) unique to SkitoSnack 2.0. The shared metabolites list is available in S1 File. The color labels above the diagram indicate the unique and common metabolites identified in the bovine blood and SkitoSnack 2.0-fed groups. The diagram was made using the BioVenn web application.

### Reproductive performance of *Ae. aegypti* fed on SkitoSnack 2.0

**Fig 6:**
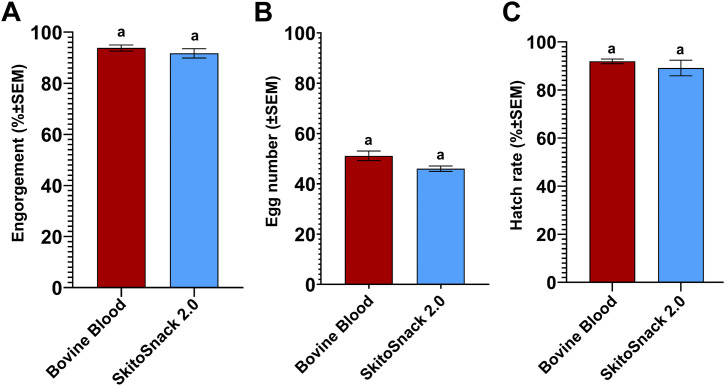
SkitoSnack 2.0 can replace vertebrate blood in *Ae. aegypti* culture. Reproductive performance of female *Ae. aegypti* fed on bovine blood or SkitoSnack 2.0 **(A)** Mean percent engorgement rate of females offered bovine blood or SkitoSnack 2.0. **(B)** Mean number of eggs laid per engorged female on bovine blood or SkitoSnack 2.0. **(C)** Mean percent larval hatch rate. An Unpaired t-test was used to determine the significant difference between diet groups. Columns with the same letters indicate no significant difference at P < 0.05. SEM stands for standard error of the mean.

We investigated the reproductive performance of female *Ae. aegypti* fed on SkitoSnack 2.0. The result from the feeding assay showed a similar engorgement rate, 93.80 ± 1.19% and 91.71 ± 1.8%, (**Fig 6A**; t_6_ = 0.967, P = 0.3713), eggs number 51 ± 1.9 and 46 ± 1.1 eggs, (**Fig 6B**; t_6_ = 2.344, P = 0.0575), and larval hatch rates,91.92 ± 0.98% and 89.15 ± 3.2%, (**Fig 6C**; t_6_ = 0.818, P = 0.4448) between female mosquitoes fed on bovine blood and SkitoSnack 2.0, respectively.

### Feeding behavior of *Ae. aegypti* in modified FlyPAD choice feeding assay

**Fig 7:**
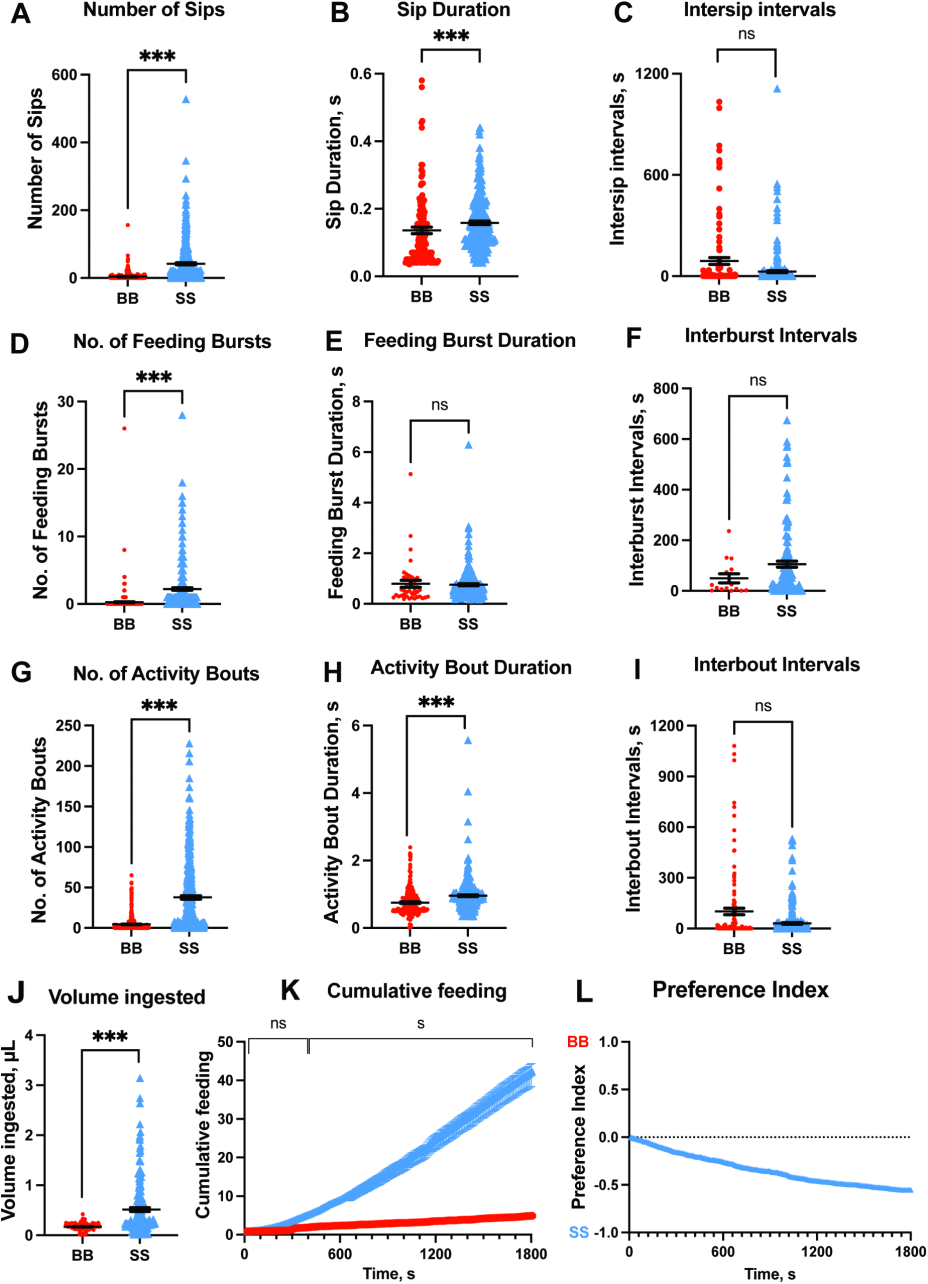
Comparison of feeding behaviors of female *Ae. aegypti* when offered bovine blood (circles in red, BB) or SkitoSnack 2.0 (triangles in blue, SS) using the FlyPAD system in choice assays, followed by quantification of the meal volume. **(A)** Number of sips.**(B)** Duration of the sips, in seconds **(s)**. **(C)** Duration of intersip intervals **(s)**. **(D)** Number of feeding bursts(**E**) Duration of each feeding burst **(s)**. **(F)** Duration of interburst intervals **(s)**. **(G)** Number of activity bouts(**H**) Duration of the activity bouts **(s)**. **(I)** Duration of interbout intervals **(s)**. **(J)** Total volume ingested by each female. **(K)** Cumulative feeding **(L)** Preference index. In panels **A**-**J**, the symbols represent results from individual mosquitoes. The horizontal lines represent mean values ± standard error of the mean (SEM). Mann–Whitney test, asterisks denote a statistical significance, where one asterisk (*) indicates P < 0.05, and two asterisks (**) indicate P < 0.01, and three asterisks (***) indicate P < 0.001, and not significant (ns) indicates P > 0.05. A two-way ANOVA was performed for cumulative feeding **(K)**, where significant differences were detected after 400 seconds (P < 0.05). The number of females per treatment in **A-I** was 384. In panel **J**, the number of females per treatment was 192.

Fig 7 shows the results of choice feeding assays from the modified FlyPAD system, followed by quantification of the meal volume to compare meal preferences.

In the choice assay, meal preference was evaluated by offering bovine blood and SkitoSnack 2.0 to the individual females (see S1 Video), and the FlyPAD system provided outputs of nine different variables corresponding to mosquito feeding behavior. The results from the FlyPAD system showed that female *Ae. aegypti* prefer SkitoSnack 2.0 over bovine blood. This is evident from the significantly greater number of sips (SS = 41.70 ± 2.98 vs. BB = 4.03 ± 0.59) (Fig 7A, U = 29480, P < 0.001), longer sip duration (SS = 0.1584 ± 0.0052 (s) vs. BB = 0.1359 ± 0.0098 (s) (Fig 7B, U = 16052, P < 0.001), similar intersip intervals durations (SS = 26.58 ± 5.84 (s) vs. BB = 89.36 ± 20.19 (s)) (Fig 7C, U = 17875, P = 0.399), higher number of feeding bursts (SS = 2.22 ± 0.18 vs. BB = 0.27 ± 0.08) (Fig 7D, U = 39417, P < 0.001), similar burst duration (SS = 0.76 ± 0.04 vs. BB = 0.79 ± 0.14) (Fig 7E, U = 3806, P = 0.399) and interburst interval (SS = 105.6 ± 12.19 vs. BB = 49.73 ± 17.82) (Fig 7F, U = 774, P = 0.274) recorded for individual females in the channel containing SkitoSnack 2.0 compared to bovine blood.

Females feeding on the SkitoSnack 2.0 channel produced a higher number of feeding bursts which resulted in a higher number of activity bouts (SS = 37.71 ± 2.13 vs. BB = 4.46 ± 0.48) (Fig 7G, U = 28447, P < 0.001), and a higher duration of activity bouts (SS = 0.96 ± 0.03 vs. BB = 0.75 ± 0.03) (Fig 7H, U = 23389, P < 0.001) with similar interbout intervals (SS = 30.58 ± 4.87 vs. BB = 101.2 ± 19.76) (Fig 7I, U = 15830, P = 0.371). Figs 7C, 7F, and 7I showed that the choice of feeding on bovine blood versus SkitoSnack 2.0 did not affect the interval duration between consecutive sips, bursts, or bouts.

The quantification of meal volume per female showed that the SkitoSnack 2.0 meal was ingested in significantly higher volume than that of bovine blood (SS = 0.5152 ± 0.0409 vs. BB = 0.1713 ± 0.0048) (Fig 7J; U = 9205, P < 0.001). The cumulative average number of sips per female was recorded every 10 s and differed significantly starting at 400 seconds (Fig 7K, F _(179, 137114)_ = 117.9, P < 0.05) until the assay endpoint at 30 min. The preference index (PI = -0.5528 ± 0.0229) (Fig 7L) also indicated that the females preferred SkitoSnack 2.0 over bovine blood.

## Discussion

Since its conception in 2017 [12], SkitoSnack has been successfully used in rearing *Ae. aegypti* mosquitoes in the laboratory [25,32,33]. However, offering this diet as a food source to other species of mosquitoes and other blood-sucking arthropods has resulted in mixed results or failure. In a 2025 study on *Culex pipiens* fed on SkitoSnack compared to blood-fed mosquitoes, Soto *et al.* reported high engorgement rates but also higher mortality and a significant reduction in egg numbers and hatch rates. Interestingly, the authors describe a variance among mosquitoes of different ages in their willingness to feed [32]. In another study, SkitoSnack was offered to 5^th^ instar bedbug nymphs, *Cimex lectularius* [25]. Only a small percentage engorged, and only 3% of the engorged bedbugs molted. Intriguingly, Gonzales and collaborators in 2024 fed SkitoSnack to both *Ae. aegypti* and *An. stephensi* and found high engorgement rates but comparatively low egg hatching rates [3]. We were unable to reproduce these results since our laboratory-reared *An. stephensi* did not imbibe on the original SkitoSnack diet (see Fig 1D). The reason for this difference is unclear. Possible variables responsible for these contrary findings are the strain of *An. stephensi* used, variances in the experimental setups, or modifications in diet preparation.

SkitoSnack 2.0 contains iron (II) fumarate as an iron source, while the original SkitoSnack recipe contains bovine hemoglobin. Iron is a crucial component for mosquito egg production and hatch rates [34–36]. However, hemoglobin at high concentrations acts as a potent phago-suppressor in *Ae. aegypti* [23]. We hypothesized that even at lower concentrations, hemoglobin acts as a phago-suppressor for *Anopheles* mosquitoes. We tested various iron sources as a replacement for hemoglobin (**see Fig 2**). Iron (II) fumarate (Ferrous fumarate) outperformed all other iron sources we tested in terms of egg numbers and hatch rates in *An. stephensi*. We used this species because it is an important urban vector of malaria in Southern Asia and the Middle East [37,38]

Interestingly, iron fumarate is widely used to treat iron deficiency anemia in humans [39]. It stands out as a good iron supplement due to its relatively high elemental iron content (33%) compared to ferrous gluconate (12%). It has good bioavailability, and better absorption with fewer side effects compared to other iron supplements [40].

SkitoSnack 2.0 can effectively support *An. stephensi* culture. The mosquito females readily engorged on it and laid a similar number of eggs as blood-fed control mosquitoes. However, we found lower hatch rates for these eggs (**see Fig 3**).

A critical question we asked next was if SkitoSnack 2.0 can support the long-term maintenance of *An. stephensi* colonies. To answer this question, we maintained an *An. stephensi* colony exclusively reared on SkitoSnack 2.0 for over five generations and compared engorgement rates, egg numbers, egg hatching rates, body weight, and wing length to colonies fed only on blood (see Fig 4). Remarkably, hatching rates after five generations on SkitoSnack 2.0 improved to numbers similar to those found in eggs from blood fed females.

The mosquito egg metabolome, the complete gamut of small molecule metabolites, is important for egg viability [41]. We performed a metabolomics study to compare groups of metabolites in eggs from SkitoSnack 2.0 and blood-fed *An. stephensi* females. The goal of this experiment was to identify critical metabolites that are correlated with reproductive success. We found that both diets resulted in similar egg metabolomes with a number of metabolites relatively enriched after feeding bovine blood (see Fig 5).Similar results were reported by Gonzales et al., who compared the egg metabolomes of eggs produced with the original SkitoSnack recipe with eggs from bovine blood-fed females [12]. These findings may provide leads on how to further improve SkitoSnack 2.0.

We then hypothesized that the new recipe can support the culture of *Ae. aegypti*. As we expected, SkitoSnack 2.0 was suitable for *Ae. aegypti* culture (see Fig 6). SkitoSnack 2.0 may also be suitable to rear other blood sucking insects as well. Future studies will support or disprove this hypothesis.

Lastly, we explored the feeding behavior and diet preference of *Ae. aegypti* when offered blood or SkitoSnack 2.0. We used the flyPAD feeding system that was originally developed for *Drosophila melanogaster* and later adapted to study mosquito feeding behavior [29,30]. When offered either diet, we found differences in feeding behavior between SkitoSnack 2.0 and bovine blood. *Ae. aegypti* fed more on SkitoSnack 2.0 and in a choice assay preferred it over blood.

In summary, we have developed a blood meal replacement diet for *Anopheles* and *Aedes* culture that produced results equivalent to vertebrate blood with the species and strains of mosquitoes we tested. The challenge for the future is to develop a diet that is better than blood.

## Supporting information

S1 File This Excel spreadsheet provides the raw data for each figure in this manuscript. (XLSX)

S2 File This word file provides supplementary figures and protocol for egg metabolomics. (DOCX)

S3 File This word file provides the standard protocol and figures for the FlyPAD feeding assay. (DOCX)

S1 Video This video file shows the feeding behavior of an individual female *Ae. aegypti* mosquitoes in FlyPAD choice feeding assay. All FlyPAD video recordings are original work by Meenakshi Berwal.
(MP4)

S1 Fig Increasing ATP concentrations have a similar engorgement in female *An. stephensi.* Shown are the mean percent engorgement after preparing modified SkitoSnack with increasing ATP concentrations. Meals were bovine blood, original SkitoSnack or modified SkitoSnack with 1X ATP, 2X ATP, and 3X ATP. A one-way ANOVA with Tukey’s multiple comparison test was used to analyse the statistical significance among different diet groups. Columns with different letters indicate a significant difference (P < 0.05).
(TIF)

S2 Fig Principal Component Analysis (PCA) scores plot based on GC-MS metabolomic profiles of samples derived from two meal formulations: Defibrinated Bovine Blood (DBB) and SkitoSnack 2.0 (SS 2.0). Each point represents an individual sample, with DBB (red) and SS 2.0 (blue) showing distinct clustering. The first two principal components (PC1 and PC2) explain 71.5% and 9.1% of the total variance, respectively.
(TIF)

S3 Fig Hierarchically clustered heat map of metabolite profiles from GC-MS-based untargeted metabolomics comparing Defibrinated Bovine Blood (DBB) and SkitoSnack 2.0 (SS 2.0) formulations. Metabolite peak areas were normalized to internal standard peak area, log₁₀-transformed, Z-score normalized, and averaged across biological replicates for each group. Rows represent individual metabolites, and columns represent treatment groups. The color gradient indicates relative abundance levels (red = higher, blue = lower Z-scores).
(TIF)

## References

[1] Organization WH. World malaria report 2023. World Health Organization. 2023.

[2] Gari T, Lindtjørn B. Reshaping the vector control strategy for malaria elimination in Ethiopia in the context of current evidence and new tools: opportunities and challenges. Malar J. 2018;17(1):454. doi: 10.1186/s12936-018-2607-8 30518395 PMC6282332

[3] Gonzales-Wartz KK, Sá JM, Lee K, Gebremicale Y, Deng B, Long CA, et al. Infectivity of Plasmodium parasites to Aedes aegypti and Anopheles stephensi mosquitoes maintained on blood-free meals of SkitoSnack. Parasit Vectors. 2024;17(1):290. doi: 10.1186/s13071-024-06364-9 38971776 PMC11227701

[4] Aldridge RL, Gibson S, Linthicum KJ. Aedes aegypti Controls AE. Aegypti: SIT and IIT-An Overview. J Am Mosq Control Assoc. 2024;40(1):32–49. doi: 10.2987/23-7154 38427588

[5] Naidoo K, Oliver SV. Gene drives: an alternative approach to malaria control?. Gene Ther. 2025;32(1):25–37. doi: 10.1038/s41434-024-00468-8 39039203 PMC11785527

[6] Njoroge TM, Hamid-Adiamoh M, Duman-Scheel M. Maximizing the Potential of Attractive Targeted Sugar Baits (ATSBs) for Integrated Vector Management. Insects. 2023;14(7):585. doi: 10.3390/insects14070585 37504591 PMC10380652

[7] Catteruccia F, Crisanti A, Wimmer EA. Transgenic technologies to induce sterility. Malar J. 2009;8 Suppl 2(Suppl 2):S7. doi: 10.1186/1475-2875-8-S2-S7 19917077 PMC2777329

[8] Gonzales KK, Hansen IA. Artificial diets for mosquitoes. Int J Environ Res Public Health. 2016;13(12). 10.3390/ijerph13121267 PMC5201408 28009851

[9] Weaver AR, Rajagopal NR, Pereira RM, Koehler PG, MacIntosh AJ, Baldwin RW, et al. Characteristics of a Spray-Dried Porcine Blood Meal for Aedes aegypti Mosquitoes. Insects. 2024;15(9):716. doi: 10.3390/insects15090716 39336684 PMC11432713

[10] da Silva Costa G, Rodrigues MMS, Silva A de AE. Toward a blood-free diet for Anopheles darlingi (Diptera: Culicidae). J Med Entomol. 2020;57(3):947–51. doi: 10.1093/jme/tjz217 31790134

[11] Marques J, Cardoso JCR, Felix RC, Santana RAG, Guerra M das GB, Power D, et al. Fresh-blood-free diet for rearing malaria mosquito vectors. Sci Rep. 2018;8(1):17807. doi: 10.1038/s41598-018-35886-3 30546023 PMC6292920

[12] Gonzales KK, Rodriguez SD, Chung H-N, Kowalski M, Vulcan J, Moore EL, et al. The Effect of SkitoSnack, an Artificial Blood Meal Replacement, on Aedes aegypti Life History Traits and Gut Microbiota. Sci Rep. 2018;8(1):11023. doi: 10.1038/s41598-018-29415-5 30038361 PMC6056539

[13] Baughman T, Peterson C, Ortega C, Preston SR, Paton C, Williams J, et al. A highly stable blood meal alternative for rearing Aedes and Anopheles mosquitoes. PLoS Negl Trop Dis. 2017;11(12):e0006142. doi: 10.1371/journal.pntd.0006142 29287072 PMC5764435

[14] Talyuli OAC, Bottino-Rojas V, Taracena ML, Soares ALM, Oliveira JHM, Oliveira PL. The use of a chemically defined artificial diet as a tool to study Aedes aegypti physiology. J Insect Physiol. 2015;83:1–7. doi: 10.1016/j.jinsphys.2015.11.007 26578294

[15] Pitts RJ. A blood-free protein meal supporting oogenesis in the Asian tiger mosquito, Aedes albopictus (Skuse). J Insect Physiol. 2014;64:1–6. doi: 10.1016/j.jinsphys.2014.02.012 24607650

[16] Griffith JS, Turner GD. Culturing Culex quinquefasciatus mosquitoes with a blood substitute diet for the females. Med Vet Entomol. 1996;10(3):265–8. doi: 10.1111/j.1365-2915.1996.tb00741.x 8887338

[17] Cosgrove JB, Wood RJ. Effects of variations in a formulated protein meal on the fecundity and fertility of female mosquitoes. Med Vet Entomol. 1996;10(3):260–4. doi: 10.1111/j.1365-2915.1996.tb00740.x 8887337

[18] Kogan PH. Substitute blood meal for investigating and maintaining Aedes aegypti (Diptera: Culicidae). J Med Entomol. 1990;27(4):709–12. doi: 10.1093/jmedent/27.4.709 2388248

[19] Dimond J, et al. A preliminary note on some nutritional requirements for reproduction in female Aedes aegypti. 1955.

[20] Lea A, et al. A preliminary note on egg production from milk-fed mosquitoes. The Ohio Journal of Science. 1955;55(1):21–2.

[21] Benedict M. The MR4 methods in Anopheles research laboratory manual. Atlanta: CDC. 2018.

[22] Das S, Garver L, Dimopoulos G. Protocol for mosquito rearing (A. gambiae). J Vis Exp. 2007;(5):221. doi: 10.3791/221 18979019 PMC2557088

[23] Gonzales KK, Tsujimoto H, Hansen IA. Blood serum and BSA, but neither red blood cells nor hemoglobin can support vitellogenesis and egg production in the dengue vector Aedes aegypti. PeerJ. 2015;3:e938. doi: 10.7717/peerj.938 26020000 PMC4435475

[24] Gonzales KK, Hansen IA. Artificial Diets for Mosquitoes. Int J Environ Res Public Health. 2016;13(12):1267. doi: 10.3390/ijerph13121267 28009851 PMC5201408

[25] Kandel Y, Mitra S, Jimenez X, Rodriguez SD, Romero A, Blakely BN, et al. Long-Term Mosquito culture with SkitoSnack, an artificial blood meal replacement. PLoS Negl Trop Dis. 2020;14(9):e0008591. doi: 10.1371/journal.pntd.0008591 32941432 PMC7523998

[26] Pilitt DR, Jones JC. A qualitative method for estimating the degree of engorgement of Aedes aegypti adults. J Med Entomol. 1972;9(4):334–7. doi: 10.1093/jmedent/9.4.334 5054499

[27] Zuharah WF, Ahbirami R, Dieng H, Thiagaletchumi M, Fadzly N. Evaluation of sublethal effects of ipomoea cairica linn. extract on life history traits of dengue vectors. Rev Inst Med Trop Sao Paulo. 2016;58:44. doi: 10.1590/S1678-9946201658044 27253746 PMC4880001

[28] Tsugawa H, Cajka T, Kind T, Ma Y, Higgins B, Ikeda K, et al. MS-DIAL: data-independent MS/MS deconvolution for comprehensive metabolome analysis. Nat Methods. 2015;12(6):523–6. doi: 10.1038/nmeth.3393 25938372 PMC4449330

[29] Henriques-Santos BM, Xiong C, Pietrantonio PV. Automated analysis of feeding behaviors of females of the mosquito Aedes aegypti using a modified flyPAD system. Sci Rep. 2023;13(1):20188. doi: 10.1038/s41598-023-47277-4 37980438 PMC10657447

[30] Itskov PM, et al. Automated monitoring and quantitative analysis of feeding behaviour in Drosophila. Nature Communications. 2014;5(1):4560. 10.1038/ncomms5560 PMC4143931 25087594

[31] Lopes G, et al. Bonsai: an event-based framework for processing and controlling data streams. Frontiers in Neuroinformatics. 2015;9:7. 25904861 10.3389/fninf.2015.00007 PMC4389726

[32] Soto A, Devlies A-S, Wauters L, Ferreira Pinto AP, Delang L. The artificial meal SkitoSnack does not support reproduction in Culex pipiens (Diptera: Culicidae) mosquitoes. J Insect Sci. 2025;25(2):17. doi: 10.1093/jisesa/ieaf022 40278044 PMC12023163

[33] Faber PA, Dorai AJAPS, Chown SL. A standardised low-cost membrane blood-feeder for Aedes aegypti made using common laboratory materials. PeerJ. 2022;10:e14247. doi: 10.7717/peerj.14247 36325181 PMC9620972

[34] Rivera-Pérez C, Clifton ME, Noriega FG. How micronutrients influence the physiology of mosquitoes. Curr Opin Insect Sci. 2017;23:112–7. doi: 10.1016/j.cois.2017.07.002 29129275 PMC5695569

[35] Zhou G, Kohlhepp P, Geiser D, Frasquillo MDC, Vazquez-Moreno L, Winzerling JJ. Fate of blood meal iron in mosquitoes. J Insect Physiol. 2007;53(11):1169–78. doi: 10.1016/j.jinsphys.2007.06.009 17689557 PMC2329577

[36] Geiser DL, Thai TN, Love MB, Winzerling JJ. Iron and Ferritin Deposition in the Ovarian Tissues of the Yellow Fever Mosquito (Diptera: Culicidae). J Insect Sci. 2019;19(5):11. doi: 10.1093/jisesa/iez089 31606748 PMC6790249

[37] Liu Q, Wang M, Du Y-T, Xie J-W, Yin Z-G, Cai J-H, et al. Possible potential spread of Anopheles stephensi, the Asian malaria vector. BMC Infect Dis. 2024;24(1):333. doi: 10.1186/s12879-024-09213-3 38509457 PMC10953274

[38] Taylor R, Messenger LA, Abeku TA, Clarke SE, Yadav RS, Lines J. Invasive Anopheles stephensi in Africa: insights from Asia. Trends Parasitol. 2024;40(8):731–43. doi: 10.1016/j.pt.2024.06.008 39054167

[39] Santiago P. Ferrous versus ferric oral iron formulations for the treatment of iron deficiency: a clinical overview. ScientificWorldJournal. 2012;2012:846824. doi: 10.1100/2012/846824 22654638 PMC3354642

[40] Toblli JE, Cao G, Oliveri L, Angerosa M. Effects of iron polymaltose complex, ferrous fumarate and ferrous sulfate treatments in anemic pregnant rats, their fetuses and placentas. Inflamm Allergy Drug Targets. 2013;12(3):190–8. doi: 10.2174/18715281113129990040 23547731

[41] Prasad A, Sreedharan S, Bakthavachalu B, Laxman S. Eggs of the mosquito Aedes aegypti survive desiccation by rewiring their polyamine and lipid metabolism. PLoS Biol. 2023;21(10):e3002342. doi: 10.1371/journal.pbio.3002342 37874799 PMC10597479

