# Supplementary material for: SkitoSnack 2.0 - A Bloodmeal Alternative for Anopheles and Aedes Mosquitoes
Source: PLoS Negl Trop Dis. 2026 Apr 17;20(4):e0014188. doi: 10.1371/journal.pntd.0014188 (PMC13089749; doi:10.1371/journal.pntd.0014188)
Supplement: S2 File — (DOCX) [file pntd.0014188.s002.docx]

**SkitoSnack 2.0 - a Bloodmeal Alternative for *Anopheles* and *Aedes* Mosquitoes**

**Supplementary Information**

Anjali Karki^1^, Hailey A. Luker^1^, Naga Narendra Reddy Potlapalli^2^, F. Omar Holguin^2^, Meenakshi Berwal^3^, Patricia V. Pietrantonio^3^, and Immo A. Hansen^1*^

**Affiliations:**

*^1^ Department of Biology, New Mexico State University, Las Cruces, NM, United States*

*^2^Department of Plant and Environmental Sciences, New Mexico State University, Las Cruces, NM, United States*

*^3^Department of Entomology, Texas A&M University, College Station, TX, United States*

*^3^Texas A&M AgriLife Research and Extension Center, Lubbock, TX, United States*

* Author to whom correspondence should be addressed.

- **Guide:**
- **Fig S1.** **Increasing ATP concentration was tested as an engorgement-enhancing agent in female *An. stephensi*.**
- **Supplementary protocol for mosquito egg metabolomics**.
- **Fig S2. Principal Component Analysis (PCA) scores plot based on GC-MS metabolomic profiles of samples derived from two meal formulations: Defibrinated Bovine Blood (DBB) and SkitoSnack 2.0 (SS 2.0).**
- **Fig S3.** **Hierarchically clustered heat map of metabolite profiles from GC-MS-based untargeted metabolomics comparing Defibrinated Bovine Blood (DBB) and SkitoSnack 2.0 (SS 2.0) formulations.**


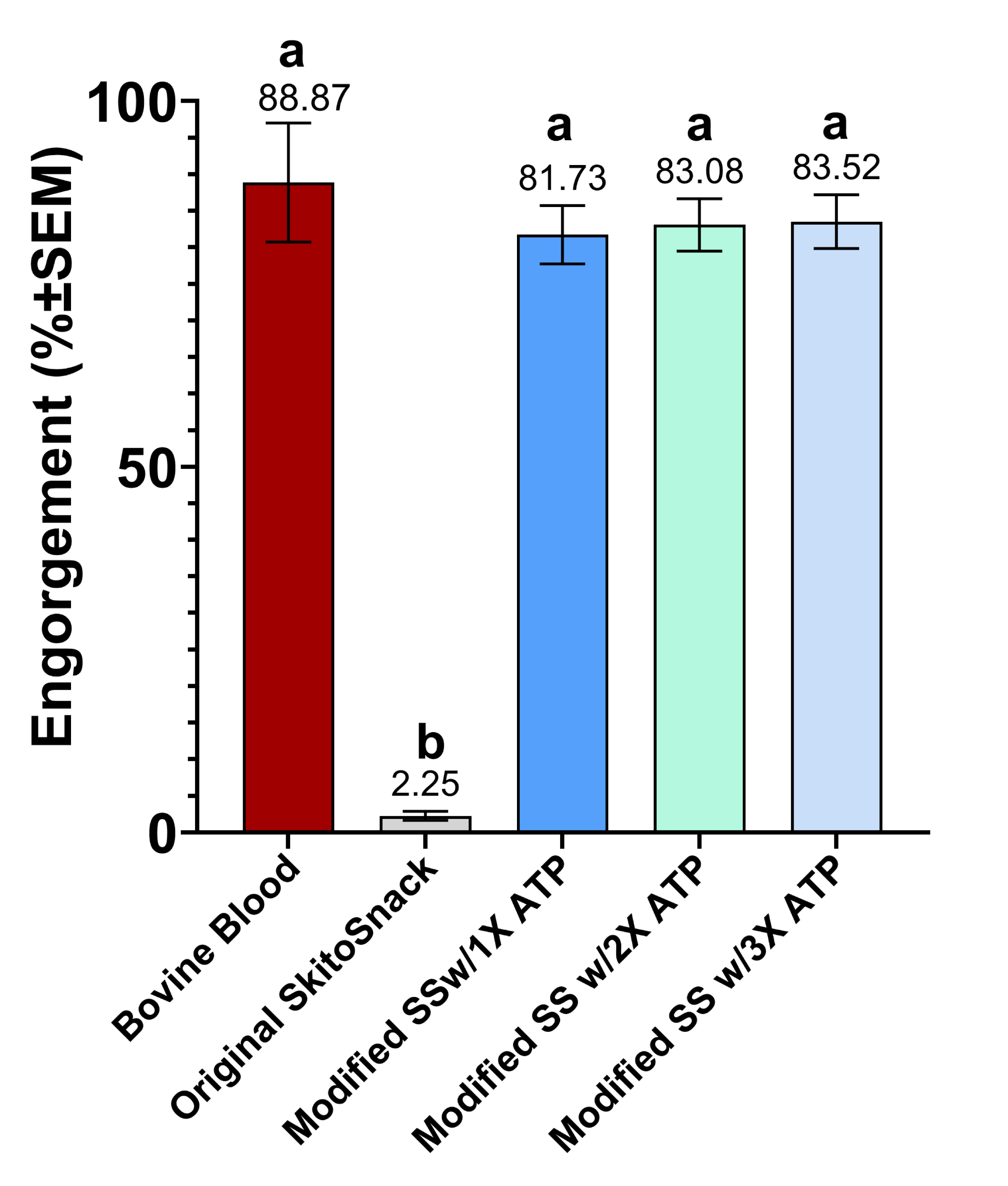


**Fig S1. Increasing ATP concentrations have a similar engorgement in female *An. stephensi*.** Shown are the mean percent engorgement after preparing modified SkitoSnack with increasing ATP concentrations. Meals were bovine blood, original SkitoSnack or modified SkitoSnack with 1X ATP, 2X ATP, and 3X ATP. A one-way ANOVA with Tukey’s multiple comparison test was used to analyse the statistical significance among different diet groups. Columns with different letters indicate a significant difference (P < 0.05)*.*

**Supplementary Protocol**

**Protocol:** Mosquito egg metabolomics

**Materials and Methods**

Mosquito eggs’ total metabolites were extracted and analyzed as described by Gonzales et al.[1] with a few modifications. Eggs were collected from two cohorts of *Anopheles stephensi* mosquitoes, (1) *Anopheles* fed on defibrinated bovine blood, and (2) *Anopheles* fed on SkitoSnack 2.0 using a membrane feeder. Approximately 8-10 mg of eggs were weighed and collected into 1.7 mL microcentrifuge tubes. These eggs were then homogenized for 15 seconds using 0.5 mL of sterile glass beads and 0.5 mL chloroform: methanol: water (10:3:1 v/v/v) in a Precellys 24 homogenizer (Bertin Instruments, US). This step was repeated three times, each time the supernatant was collected in a 1.7 mL microcentrifuge tube. The supernatant was dried using a vacuum centrifuge (Eppendorf, US) for over three hours. The next day, 3 μL of 20 mg/ ml ribitol in pyridine, 5 μL of 1 mg/ ml C4-C24 Even Carbon Saturated FAMEs (Sigma-Aldrich, US), and 45 μL of 20 mg/ ml methoxyamine HCl: Pyridine was added to the dried samples and placed in an incubating shaker for 1.5 hours at 37 °C. After that, 45 μL of MSTFA was added to each sample, and the samples were incubated in a shaking incubator for another half an hour at 37 °C. These samples were then centrifuged, and the supernatant was transferred to 1.5 mL amber glass vials (Agilent, US) for chromatographic separation and mass analysis. Metabolites were separated with a Varian Saturn 2000 (Varian Inc., Walnut Creek, CA, USA), and analyzed with a leco pegasus high throughput time-of-flight mass spectrometer (TOF-MS) (Leco, US).

Total ion chromatograms were deconvoluted with ChromaTof 4.41 (Leco, US), and metabolites were searched against the Fiehn library. These metabolites were identified based on retention time (min) and mass-to-charge ratio (m/z). Unique ions were selected by a signal-to-noise (S/N) threshold of 50 and quantified by peak area. Spectral data were aligned by the internal standard (ribitol) in MS-DIAL (Noble Foundation, US). Peak areas were normalized to sample weight and log_2_-transformed for computational analysis.All the data were uploaded to MS-DIAL for statistical analysis.


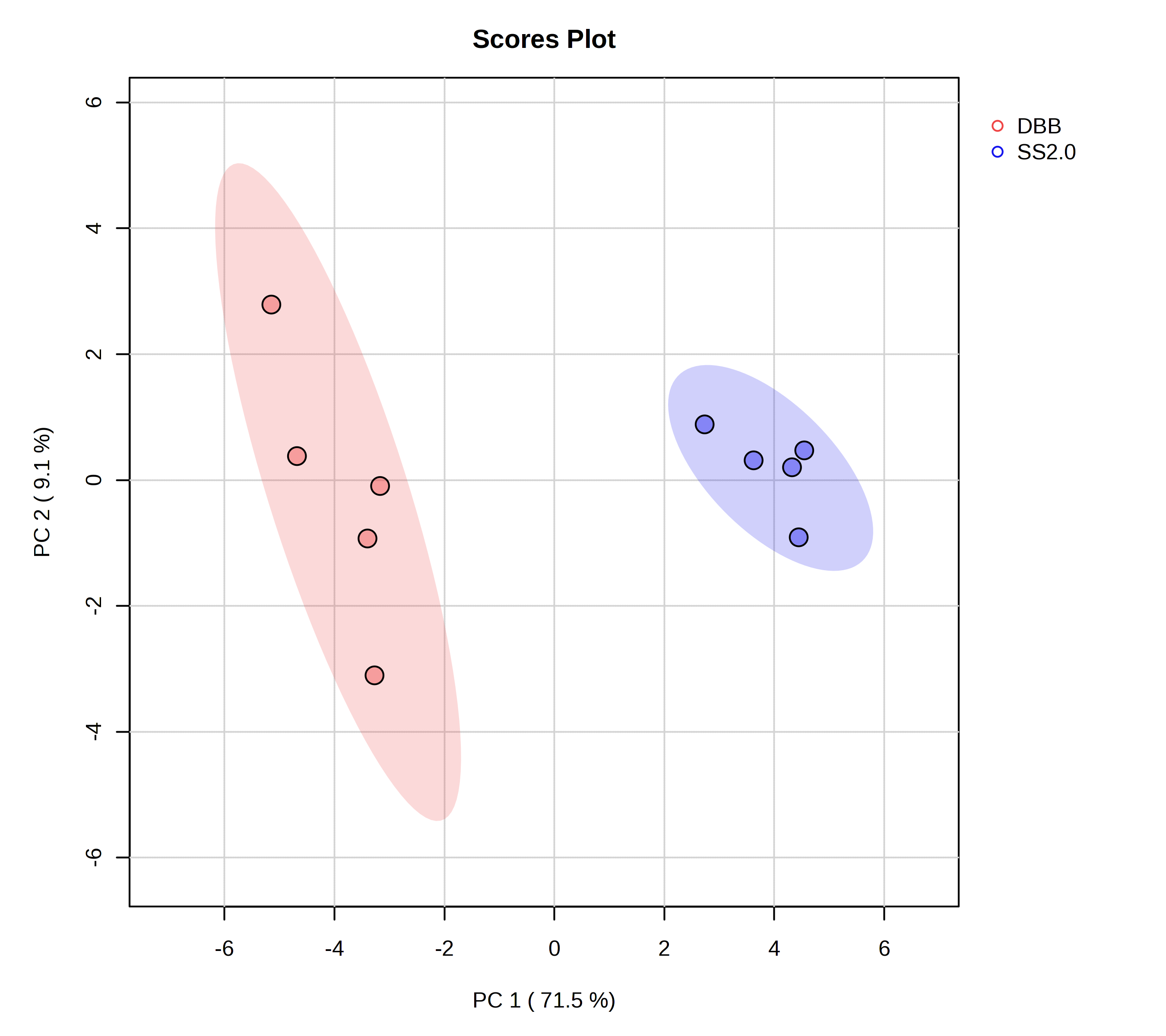


**Fig S2. Principal Component Analysis (PCA) scores plot based on GC-MS metabolomic profiles of samples derived from two meal formulations: Defibrinated Bovine Blood (DBB) and SkitoSnack 2.0 (SS 2.0).** Each point represents an individual sample, with DBB (red) and SS 2.0 (blue) showing distinct clustering. The first two principal components (PC1 and PC2) explain 71.5% and 9.1% of the total variance, respectively.


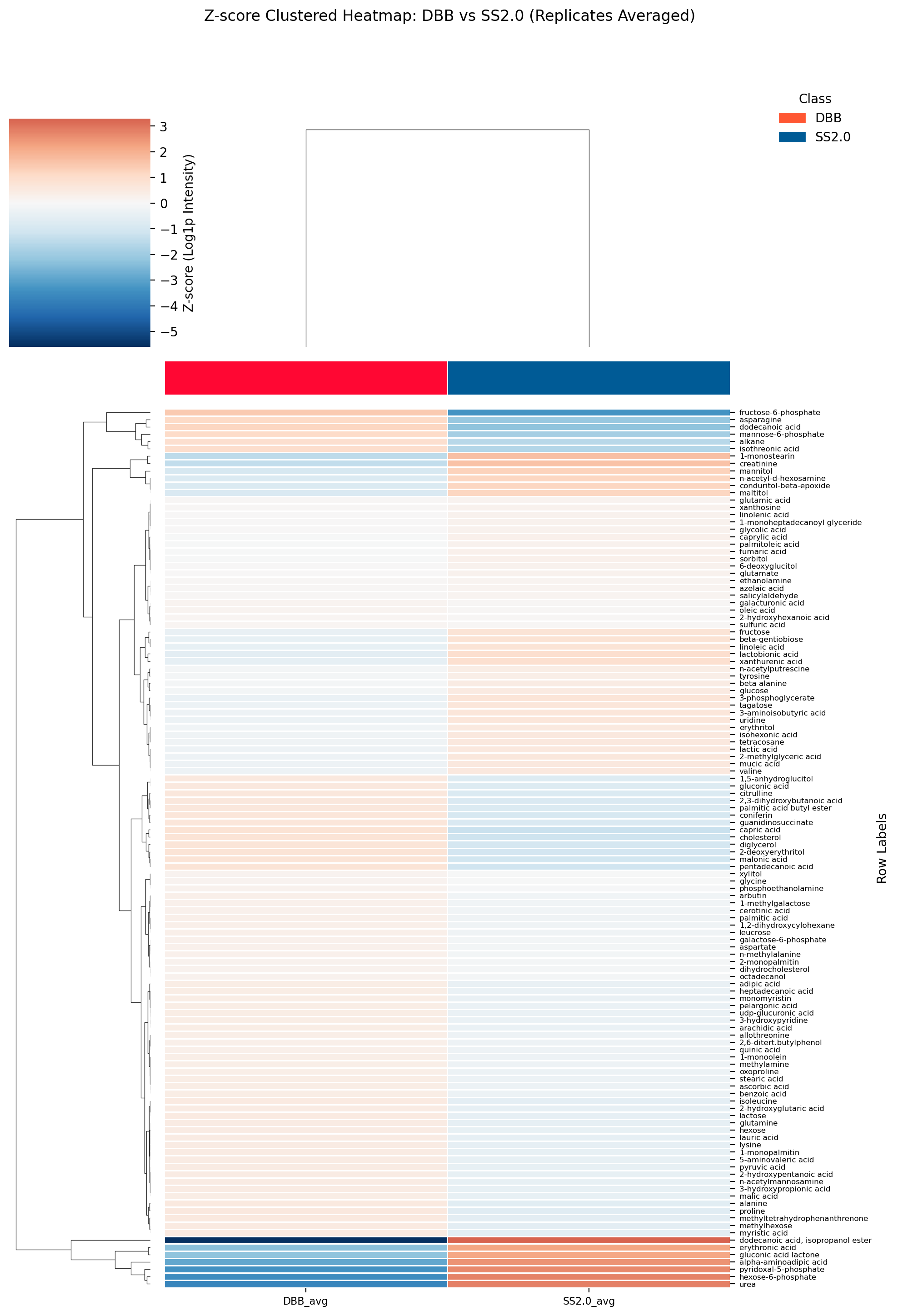


**Fig S3. Hierarchically clustered heat map of metabolite profiles from GC-MS-based untargeted metabolomics comparing Defibrinated Bovine Blood (DBB) and SkitoSnack 2.0 (SS 2.0) formulations.** Metabolite peak areas were normalized to internal standard peak area, log₁₀-transformed, Z-score normalized, and averaged across biological replicates for each group. Rows represent individual metabolites, and columns represent treatment groups. The color gradient indicates relative abundance levels (red = higher, blue = lower Z-scores).

**Reference**

1. Gonzales, K.K., et al., *The effect of SkitoSnack, an artificial blood meal replacement, on Aedes aegypti life history traits and gut microbiota.* Scientific Reports, 2018. **8**(1): p. 11023.
