# Supplementary material for: SkitoSnack 2.0 - A Bloodmeal Alternative for Anopheles and Aedes Mosquitoes
Source: PLoS Negl Trop Dis. 2026 Apr 17;20(4):e0014188. doi: 10.1371/journal.pntd.0014188 (PMC13089749; doi:10.1371/journal.pntd.0014188)
Supplement: S3 File — (DOCX) [file pntd.0014188.s003.docx]

**SkitoSnack 2.0 - a Bloodmeal Alternative for *Anopheles* and *Aedes* Mosquitoes**

**Supplementary Protocol**

Anjali Karki^1^, Hailey A. Luker^1^, Naga Narendra Reddy Potlapalli^2^, F. Omar Holguin^2^, Meenakshi Berwal^3^, Patricia V. Pietrantonio^3^, and Immo A. Hansen^1*^

**Affiliations:**

*^1^ Department of Biology, New Mexico State University, Las Cruces, NM, United States*

*^2^Department of Plant and Environmental Sciences, New Mexico State University, Las Cruces, NM, United States*

*^3^Department of Entomology, Texas A&M University, College Station, TX, United States*

*United States*

*^3^Texas A&M AgriLife Research and Extension Center, Lubbock, TX, United States*

* Author to whom correspondence should be addressed.

- **Guide**
- **Supplementary protocol for Feeding behavioral bioassays**
- **Fig S4.** **Comparison of feeding behaviors of females of *Aedes aegypti* (Liverpool strain) when offered bovine blood (circles in red, BB) or SkitoSnack 2.0 (triangles in blue, SS) using the FlyPAD system in non-choice assays.**

**Protocol:** Feeding behavioral bioassays

**Materials and Methods**

**Mosquito rearing**

Eggs of the Liverpool strain of *Aedes aegypti* (L.) originating from females have been fed bovine blood from several generations that were hatched in an autoclaved mix of water added with ground fish food (Tetra, Blacksburg, VA). Aquatic larval stages were reared in 1-litre trays, and ground fish food was provided for their larval development. Larvae were kept at low densities to ensure homogeneous large-sized adults [1, 2]. Pupae were collected in Styrofoam cups and placed in cages (30.5 cm × 30.5 cm × 30.5 cm (BioQuip Products Inc.) at 28°C and 70-80% relative humidity, 16 h light:8 h dark cycle in an incubator (Percival Scientific Inc.). Adults were provided with 10% (w/v) sucrose solution in a covered cup with partially soaked cotton wicks.

For all feeding behavior bioassays, 7-14-day-old females were used. These females were sugar-starved for 24 h before experiments by replacing the sucrose solution with water.

**Meal preparation for non-choice and choice assays**

For preparing 1 mL SkitoSnack 2.0 solution, 0.232 g of SkitoSnack 2.0 powder was weighed in a microtube and solubilized in 1 mL Milli-Q water, then vortexed vigorously. The solution was then incubated at 37°C for 3-5 min in a water bath. After incubation, the meal was vortexed again to ensure it was properly dissolved. Meals were used within 1-2 h of preparation.

**For non-choice feeding assays**, the SkitoSnack 2.0 meal was prepared by adding 0.002% (w/v) fluorescein, and the bovine blood (HemoStat Laboratories, Dixon, CA, USA) meal was freshly prepared by adding 1 mM ATP and 0.002% (w/v) fluorescein as final concentrations. The ATP solution was prepared as previously described [3]. To allow quantification of the meal volume ingested, a 0.2% (w/v) stock aqueous solution of fluorescein sodium salt (VWR Radnor, PA, USA, 0681-100G) was prepared and wrapped in aluminum foil to avoid light exposure [4, 5].

**Choice assays** were followed or not by meal volume quantification. For choice feeding assays in which the meal volume was not quantified, the meals were prepared as indicated above but without adding fluorescein.

For the choice feeding assays followed by meal volume quantification, the latter was achieved by alternating the addition of fluorescein to either SkitoSnack 2.0 or the blood meal in the wells of the arena in consecutive assay replications. In each replication, this allowed quantification of one type of meal offered to the same female mosquito using only one fluorescent dye. In the first replication, one of the meals (e.g., SkitoSnack 2.0) was supplemented with 0.002% (w/v) fluorescein while the other meal (e.g., bovine blood with 1 mM ATP) was not supplemented with the fluorescent tracer. Then in the second replication, fluorescein was used in the other meal type (e.g. bovine blood with 1 mM ATP and 0.002% (w/v) fluorescein, while the SkitoSnack 2.0 was not added fluorescein). Eight total replicates were performed, resulting in four replicates with meal quantification for each meal type.

**FlyPAD bioassays**

The modified flyPAD system was used for automated analysis of feeding behaviors of females of *Ae. aegypti* (Henriques-Santos et al., 2023). The flyPAD system consists of twelve chambers, each with four arenas, and each arena has two wells. The chambers were placed on two slide warmers (Barnstead/Lab-Line, USA, and Premiere, XH-2001, C&A Scientific Co., Inc.) maintained at 39 °C for all behavioral bioassays. In all assays 3 μL of meals were pipetted per well. In choice assays, each well of the arena received one of the meals, and in non-choice assays, both wells of the same arena received the same meal. After pipetting three microliters of meals to the wells, 7 to14-day-old-females were anesthetized for 30 s using CO_2,_ and one female was placed in each arena. The feeding behavior was recorded for 30 min.

The Bonsai data stream processing package was used to acquire streamed capacitance data [6]. A Blackfly camera (FLIR Integrated Imaging Solutions, Inc., BFS-U3-16S2C-CS) captured video recordings of a sample arena. The MATLAB (MathWorks Inc., Portola Valley, CA, USA) was used for all signal processing and data analysis. The flyPAD system recorded nine feeding behavioral variables, including number of sips (contact of proboscis with food), sip duration (period of contact of proboscis with meal) and their intervals; number, duration and intervals of feeding bursts (three or four consecutive sips), feeding bouts (three or four consecutive bursts), etc.[7]. In addition, the software provided the cumulative feeding, which includes the average number of sips performed by all females, recorded every 10 s through the 30 min of recordings. For choice assays, the cumulative preference index (PI) was calculated using the formula: PI = (n_BB_ - n_SS_) / (n_BB_ + n_SS_), where n_BB_ and n_SS_ represent the cumulative number of sips measured for bovine blood and SkitoSnack 2.0 meal, respectively, at consecutive 10 s intervals, for the 30 min of recordings [7].

**Quantification of meal volume ingested**

The fluorescein quantification method was used to estimate the total volume of bovine blood or SkitoSnack 2.0 ingested by females of *Ae. aegypti* during feeding behavioral bioassays, as described in [4],[5],[3]. For preparing a standard curve, twenty μL of meals either SkitoSnack 2.0 with 0.002% fluorescein or bovine blood containing 0.002% fluorescein and 1 mM ATP was added to 380 μL of Milli-Q water; serial dilutions were performed at a 1:2 dilution rate (200 μL final volume for each concentration). For each dilution, 100 μL of the solution was transferred to 1.2 mL tubes containing two 2.8 mm ceramic beads and one female mosquito (sugar starved for 24 h) was added in each tube and homogenized for 30 s using a TissueLyser II (QIAGEN, Aarhus, Denmark).

Upon completion of the 30 min feeding assays, females were immediately anesthetized using CO_2_, transferred individually to 1.2 mL eight strips polypropylene cluster tubes (Corning Inc., 4408) to which two 2.8 mm ceramic beads (VWR, 19-646) and Milli-Q water (100 μL) were added; and the tubes were closed with eight-cap polyethylene strips (Corning Inc., 4418). Samples were stored at - 20 °C in the dark for 48 h. For reading their fluorescence intensity, samples were thawed and homogenized for 30 s as described above for the standard curve tubes. Mosquito samples from the feeding assays and the standard curve solutions were simultaneously read for fluorescence intensity. For this, 20 μL of homogenized samples or standard curve solutions were pipetted to a 96-well black/clear bottom plate (Greiner, 655090) with 180 μL Milli-Q water. The Clariostar plate reader (BMG Labtech, Ortenberg, Germany) was used to detect the fluorescence intensity at wavelengths 485/520 nm excitation/emission. Then the total volume of the ingested meal was calculated as the ratio of the relative fluorescence units (RFU) of each female homogenate sample and the standard curve slope (RFU/μL).

**Statistical analysis**

Data from all feeding behavior bioassays were analyzed, and graphs were plotted using GraphPad Prism v10 (GraphPad Software Inc., San Diego, CA, USA). Variables measured were analyzed by a nonparametric Mann-Whitney test, followed by descriptive statistics, and results were presented as mean ± standard error of the mean (SEM). The cumulative feeding behavioral data were analyzed using a two-way ANOVA, including analysis of repeated measures and multiple comparisons of means between groups at each time point.

**Video Monitoring:**

All bioassay video monitoring was done for a sample arena (well numbers 57-56). Notice the excretion of drops of fluid after the female fed on SkitoSnack 2.0.

**Camera view of the flyPAD arena:**

Well No. 57 SkitoSnack 2.0

Well No. 56 Bovine Blood

Arena

56

57


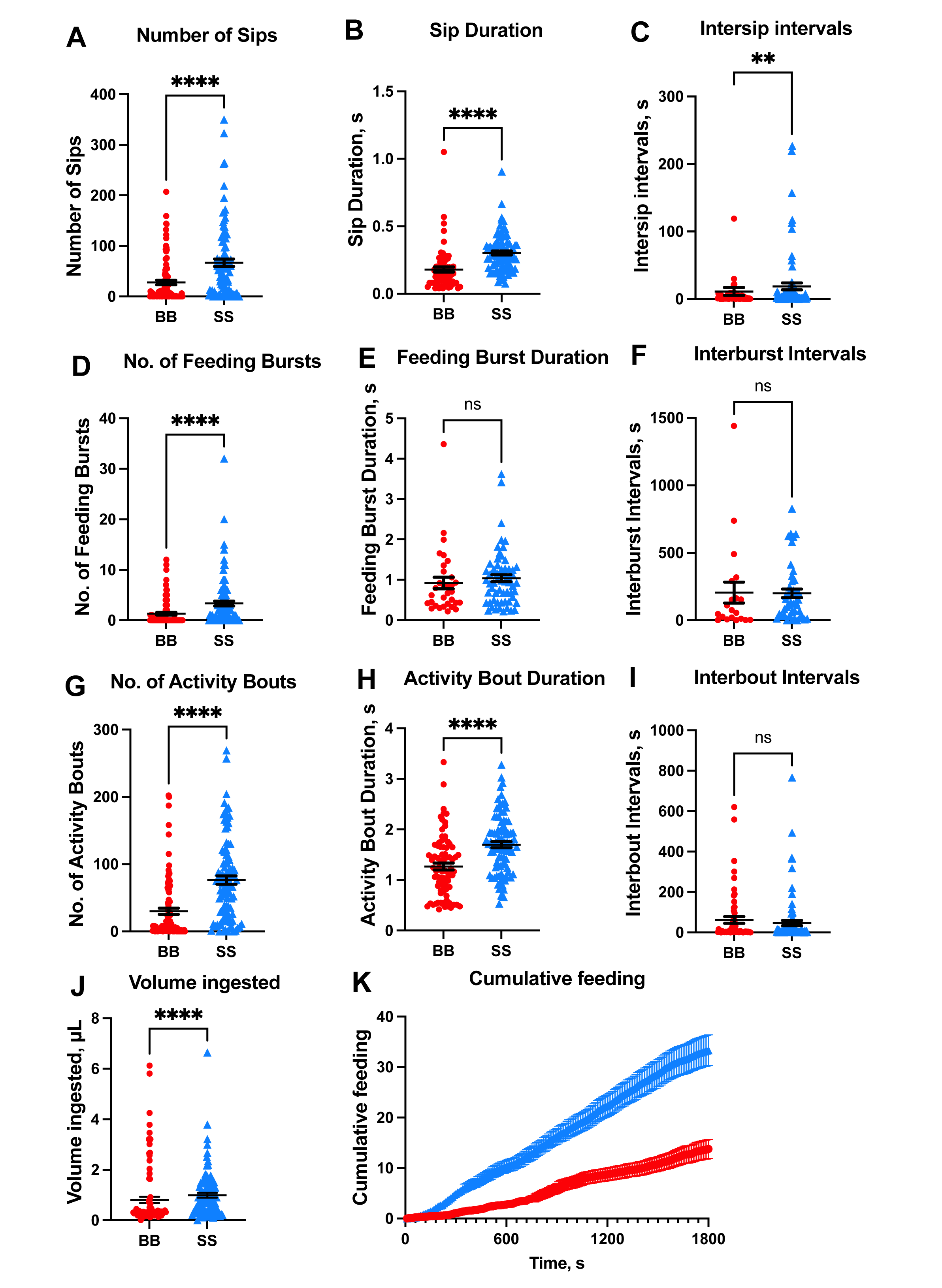


**Fig S4.** **Comparison of feeding behaviors of females of *Aedes aegypti* (Liverpool strain) when offered bovine blood (circles in red, BB) or SkitoSnack 2.0 (triangles in blue, SS) using the FlyPAD system in non-choice assays.** (**A**) Number of sips. (**B**) Duration of the sips, in seconds (s). (**C**) Duration of intersip intervals (s). (**D**) Number of feeding bursts. (**E**) Duration of each feeding burst (s). (**F**) Duration of interburst intervals (s). (**G**) Number of activity bouts (**H**) Duration of the activity bouts (s). (**I**) Duration of interbout intervals (s). (**J**) Total volume ingested by each female. (**K**) Cumulative feeding. In panels **A**-**J**, symbols represent outputs from individual mosquitoes (n per treatment = 96), horizontal lines in panels **A**-**J** represent mean values ± standard error of the mean (SEM). Mann–Whitney test, asterisks denote a statistical significance, where one asterisk (*) indicates *P*< 0.05, and two asterisks (**) indicate *P* < 0.01, and three asterisks (***) indicate *P* < 0.001, four asterisks (****) indicate *P* < 0.0001, and ns indicates not significant differences, *P*> 0.05.

**References:**

1. Price, D.P., et al., *Small mosquitoes, large implications: crowding and starvation affects gene expression and nutrient accumulation in Aedes aegypti.* Parasites & vectors, 2015. **8**: p. 1-14.

2. Shiao, S.-H., et al., *Juvenile hormone connects larval nutrition with target of rapamycin signaling in the mosquito Aedes aegypti.* Journal of insect physiology, 2008. **54**(1): p. 231-239.

3. Henriques-Santos, B.M., C. Xiong, and P.V. Pietrantonio, *Automated analysis of feeding behaviors of females of the mosquito Aedes aegypti using a modified flyPAD system.* Scientific Reports, 2023. **13**(1): p. 20188.

4. Jové, V., et al., *Feeding and quantifying animal-derived blood and artificial meals in Aedes aegypti mosquitoes.* JoVE (Journal of Visualized Experiments), 2020(164): p. e61835.

5. Venkataraman, K., V. Jové, and L.B. Duvall, *Size quantification of blood and sugar meals in Aedes aegypti mosquitoes.* Cold Spring Harbor Protocols, 2022. **2022**(6): p. pdb. prot107862.

6. Lopes, G., et al., *Bonsai: an event-based framework for processing and controlling data streams.* Frontiers in neuroinformatics, 2015. **9**: p. 7.

7. Itskov, P.M., et al., *Automated monitoring and quantitative analysis of feeding behaviour in Drosophila.* Nature communications, 2014. **5**(1): p. 4560.
